# Reconstruction and analysis of a large-scale binary Ras-effector signaling network

**DOI:** 10.1186/s12964-022-00823-5

**Published:** 2022-03-04

**Authors:** Simona Catozzi, Camille Ternet, Alize Gourrege, Kieran Wynne, Giorgio Oliviero, Christina Kiel

**Affiliations:** 1 Systems Biology Ireland, School of Medicine, University College Dublin, Belfield, Dublin 4, Ireland; 2 UCD Charles Institute of Dermatology, School of Medicine, University College Dublin, Belfield, Dublin 4, Ireland; 3 Conway Institute of Biomolecular and Biomedical Research, University College Dublin, Dublin 4, Ireland; 4 Present Address: Department of Molecular Medicine, University of Pavia, 27100 Pavia, Italy

**Keywords:** Pathway reconstruction, Ras, Effectors, Signaling pathways, Network hubs, Crosstalk, Feedbacks

## Abstract

**Background:**

Ras is a key cellular signaling hub that controls numerous cell fates via multiple downstream effector pathways. While pathways downstream of effectors such as Raf, PI3K and RalGDS are extensively described in the literature, how other effectors signal downstream of Ras is often still enigmatic.

**Methods:**

A comprehensive and unbiased Ras-effector network was reconstructed downstream of 43 effector proteins (converging onto 12 effector classes) using public pathway and protein–protein interaction (PPI) databases. The output is an oriented graph of pairwise interactions defining a 3-layer signaling network downstream of Ras. The 2290 proteins comprising the network were studied for their implication in signaling crosstalk and feedbacks, their subcellular localizations, and their cellular functions.

**Results:**

The final Ras-effector network consists of 2290 proteins that are connected via 19,080 binary PPIs, increasingly distributed across the downstream layers, with 441 PPIs in layer 1, 1660 in layer 2, and 16,979 in layer 3. We identified a high level of crosstalk among proteins of the 12 effector classes. A class-specific Ras sub-network was generated in CellDesigner (.xml file) and a functional enrichment analysis thereof shows that 58% of the processes have previously been associated to a respective effector pathway, with the remaining providing insights into novel and unexplored functions of specific effector pathways.

**Conclusions:**

Our large-scale and cell general Ras-effector network is a crucial steppingstone towards defining the network boundaries. It constitutes a ‘reference interactome’ and can be contextualized for specific conditions, e.g. different cell types or biopsy material obtained from cancer patients. Further, it can serve as a basis for elucidating systems properties, such as input–output relationships, crosstalk, and pathway redundancy.

**Graphical abstract:**

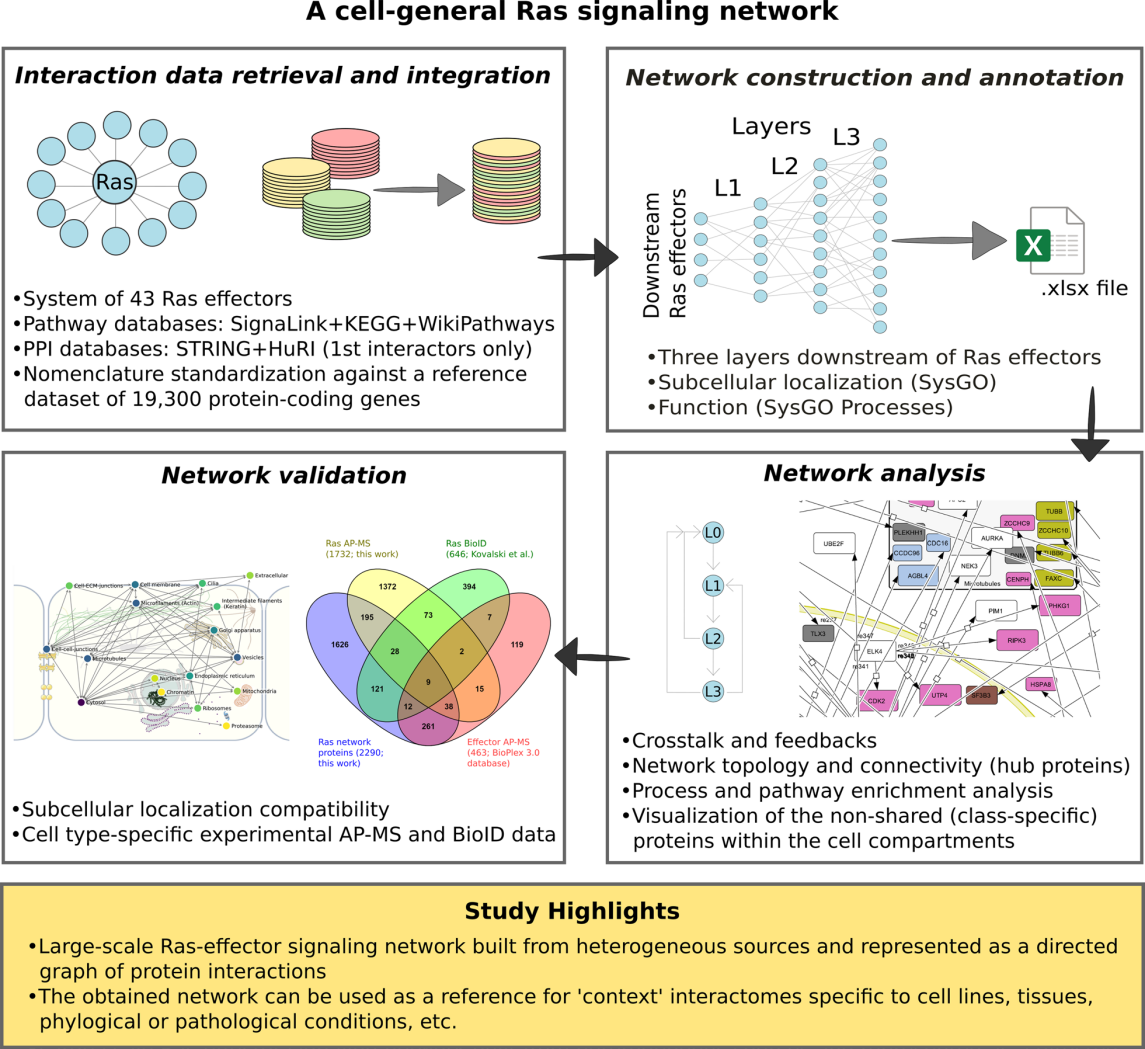

**Video Abstract**.

**Supplementary Information:**

The online version contains supplementary material available at 10.1186/s12964-022-00823-5.

## Background

Since their discovery in the 1960s, the three oncoproteins of the Ras family—HRAS, NRAS, and KRAS—have been in the spotlight of cancer research [1]. Many different tumors harbor mutations in these proteins that play a role in cancer initiation and progression, including altered metabolism, circumvention of the immune system repression, and metastasis [2]. Ras proteins can coordinate and dispatch various cellular processes of the signal transduction machinery, functioning as signaling hubs [3]. Their centrality has important repercussions on the network’s plasticity, and whenever they get deregulated, the result is a differential rewiring of the interactome [4, 5].

The first proteins interacting with Ras, known as Ras effectors, modulate the downstream signaling events through signaling cascades. Effectors are characterized by the presence of a Ras binding domain (RBD, a ubiquitin-like domain ββαββαβ) that permits the Ras-effector interaction. The number of Ras effectors has been growing considerably in the last decades [6–8], and our previous work has identified 56 RBD-containing proteins as potential Ras effectors and classified them according to their contribution to the assembly of Ras-effector complexes in the colon context [9] and in 29 human tissues [10]. In particular, our in silico computational simulations highlighted 41 effectors, of the abovementioned 56, that are predicted to be key players for Ras signaling for a specific context, stimulation, or condition [10]. More research, however, is needed to experimentally validate those predictions and confirm if such physical interactions biochemically occur.

Ras binds to its effector proteins in a mutually exclusive fashion and previous studies demonstrated that (context-specific) differential protein expression can drive differential pathway activation and functional diversity [11]. Moreover, tissue-specific concentrations and context-specific apparent binding affinities can alter the specific Ras-effector complex formations [9], making these parameters critical to establish the outcome of the competition for binding to Ras. As a consequence, effector recruitment to the plasma membrane (due e.g. to a stimulus) is an additional factor to consider. This typically endorses a selective increase of the effector local concentration, thus an enhanced interaction with Ras, which is especially ‘advantageous’ for those effectors that generally have a weak affinity for Ras [10].

The mechanism of interaction competition allows for response specificity through network rewiring, that drives the signal transduction outcome. This process requires active decision making, checkpoints authorization and information processing, which is exerted via positive/negative feedback regulation, protein scaffolding, crosstalk with other signaling molecules, etc. Despite being the foundational unit for biological systems analysis, a signaling pathway is hard to delimit—both in terms of its extent and pathway members,—as a result, its definition is far from being set and standard [12]. The challenges around pathway boundary and constraints echo the betimes contradictory knowledge deposited in different protein–protein interaction (PPI) databases [13]. This adds up to the ambiguity derived from the nomenclature that is not yet univocal, and the heterogeneity of pathway data, annotation and database features that all can depend on the scope for which each database was compiled and developed for [14].

Pathway reconstruction for physiologically-relevant analyses (e.g. [15, 16]) requires the inclusion of a sufficient number of molecular species and reactions, that must be neither too small nor too large, to the detriment of limited model interpretability and use, or inadequate compound interconnectivity. The identification of the proteins participating in the signaling network downstream a hub such as Ras is definitely a major challenge. The most well-studied Ras-mediated pathways are the mitogen-activated protein kinase (MAPK) and the phosphatidylinositol 3-kinase (PI3K) cascades, triggered by the effectors RAF and PI3 kinases, respectively. Beside these two effectors, at least 10 others have been detected [6]. However, the limited knowledge about less-studied effectors has implications for both drug development and therapeutic target discovery. A recent study experimentally recognized some new efficient Ras-binding proteins (including RIN1, RIN2, RADIL, and RALGDS-family proteins), by performing oncoRas-dependent PPI subnetworks and genetic interactions mapping, in different cell lines [17]. These findings support the need to bring ‘alternative’ Ras effectors in the spotlight beyond the classic kinases RAF and PI3K, whose poorly-understood functions may prove to be crucial in particular contexts, microenvironments or diseases [4, 7].

In this study, we generated a large-scale Ras-effector signaling network, characterized by a wide-spread crosstalk downstream the different Ras effector pathways. Our (cell general) network represents a ‘reference interactome’ that can be refined and contextualized for specific tissues and cell types. Here, we tested it in the intestine and colorectal cancer context, focusing on Caco2-derived data issued from published BioID (proximity-dependent biotinylation identification) [18] and in-house AP-MS (affinity purification coupled with mass spectrometry) measurements.

## Materials and methods

### High-confidence Ras effectors and associated pathways

Previously we described 56 effectors that contain a structural domain (RBD, i.e. Ras binding domain) with the potential ability to bind to Ras oncoproteins [9]. Of those, 43 were predicted to be high-confidence effectors, because they (i) either have a high affinity in complex with RasGTP via their RBD or (ii) are predicted to be in significant amount in complex with RasGTP using a model that includes both, binding of the RBD to Ras (with generally weak binding affinities) and binding of additional domains in effectors to the plasma membrane (recruitment of proteins to the plasma membrane via two domains is strongly enhancing the complex formation, which is referred to as “piggy-back mechanism” by Kholodenko et al. [19]; comprising the effectors from the ‘efficient binder’ groups 1 and 2 in [10]). This network is centered on the oncoproteins HRAS, KRAS, and NRAS and not on other Ras family members like MRAS, RAP1, etc., although some of the effectors considered in this work have also been shown to bind to other Ras family members—sometimes even with higher binding affinity [20, 21].

The set of 43 effectors considered in this study (grouped into 12 associated functional pathway classes) are the following—(1) RAF-MEK-ERK signaling, with the effectors ARAF, BRAF, and RAF1; (2) PI3K-AKT signaling, with the effectors PIK3CA, PIK3CB, PIK3CD, PIK3CG, PIK3C2B, and PIK3C2A; (3) RalGEF-Ral-PLD-Sec5 signaling, with the effectors RALGDS, RGL1, and RGL2; (4) Afadin-Actin-cadherin signaling, with the effector AFDN; (5) PLCε-DAG-IP_3_ signaling, with the effector PLCE1; (6) RIN-ABL-RAB signaling, with the effectors RIN1, RIN2, RIN3, and SNX27; (7) RhoGEF-RAC-PAK signaling, with the effectors TIAM1, ARHGAP20, ARAP1, ARAP2, and DGKQ; (8) RASSF-MST-Hippo signaling, with the effectors RASSF1, RASSF2, RASSF3, RASSF4, RASSF5, RASSF6, RASSF7, and RASSF8; (9) RapGEF-RAP signaling, with the effectors RAPGEF2, RAPGEF3, RAPGEF4, RAPGEF5, RAPGEF6, APBB1IP, and RAPH1; (10) Myosin-Actin signaling, with the effector MYO9B; (11) RGS-GPCR signaling, with the effector RGS12; and (12) RTK-Grb signaling, with the effectors GRB7, GRB10, and GRB14.

### Pathway and PPI databases

To reconstruct pathways and direct binary PPIs downstream of the 43 effectors, five different resources were used. The PPIs for *Homo sapiens* were retrieved from three pathway databases. We downloaded the complete dataset of interactions from SignaLink 2.0 [22] (http://signalink.org); thereof we extracted the PPIs related to our 43 Ras effectors and selected the interactions labeled either as “Directed” or “Predicted as directed”, in order to be strict—but comprehensive—about the set of first interactors of the Ras effectors. Hence, those constituted the layer 1 (L1) interactions between a source (L0 protein) and a target (L1 protein). To construct layers L2 and L3 PPIs, we iterated the database mining from the target proteins of the previous layer, then taken as sources for the following interaction (Additional file 1: Table S1). Finally, we obtained a collection of PPIs downstream Ras distributed across three layers. Moreover, we complemented our signaling network with knowledge from other pathway databases such as KEGG [23] and WikiPathways [24] whose graphical representations of the “Ras signaling pathway (*H. sapiens*)” were transformed into a list of binary (directed) interactions (like in SignaLink), to complete the former PPIs table. Still, for 28 effectors (APBB1IP, ARAP1, ARAP2, ARHGAP20, DGKQ, MYO9B, PIK3C2A, PIK3CD, PLCE1, RALGDS, RAPGEF2, RAPGEF3, RAPGEF4, RAPGEF6, RAPH1, RASSF2, RASSF3, RASSF4, RASSF5, RASSF6, RASSF7, RASSF8, RGL2, RGS12, RIN2, RIN3, SNX27, TIAM1) no pathway information was available from the databases mentioned above. Therefore, we decided to include *undirected* interactions retrieved from STRING [20] (https://string-db.org/; by setting “Experiments” and “Databases” for the active interaction sources, “Medium confidence of 0.5” for the minimum required interaction score, and “All interactors of the 1st shell”) and from the HuRI database [21] (http://www.interactome-atlas.org). Eventually, only 2 effectors (ARHGAP20 and ARAP2) were left with no known interactors, according to any of the above-mentioned databases (SignaLink, KEGG, WikiPathways, STRING, HuRI). The final interaction data is presented as an Excel file of binary PPIs, identifying a source protein and a target protein, complemented with annotations about both the proteins and their interaction (e.g. protein’s main subcellular localization, interaction type, etc.—see details further down in Materials and Methods). All the data integration and treatment were performed with the Python library *pandas*.

### Network boundary

The upper network boundary is set to include all effector proteins. This boundary follows the main purpose of the Ras-network, which is to assist in the analysis of experimental data obtained from AP-MS or BioID data on the level of Ras-effector interactions with Ras as bait.

The proteins identified in such experiments participate in larger complexes mediated by Ras. As Ras interacts with effectors in a mutually exclusive fashion, our assumption is that different Ras-mediated subcomplexes exist. These subcomplexes are expected to impact signaling pathways and cellular phenotypes. Hence, there is a strong focus on including a maximum of effector proteins as part of the upper boundary. The lower network boundary is set to include three layers. First, including three layers enables the inclusion of famous signaling cascades like RAF/MEK/ERK, PI3K/AKT/mTOR, but also RASSF10/NPM/RNF2 [25], p38MAPK-p53-survivin [26]. Second, already after three layers, the network comprises more than 10% of all human proteins (around 20 k). Including more downstream layers would have massively increased the number of network proteins. Additionally, this would shift the focus away from Ras and effectors and scatter the analysis to almost all cellular processes coordinated by Ras.

### Official gene symbols

In order to avoid duplicates in the combined dataset, the protein nomenclature (possibly inconsistent from one database to the other) was standardized against a reference set of 19,300 protein-coding genes [27]. Only three gene names (PIP3, IP3, and CCNYL3) were missing from the reference collection [27] and have been discarded from our PPI dataset, together with the related subsequent interactions (i.e. CCNYL3 interacting with PPARD, CUL1, NCOA1).

### Global expression levels of network proteins in human tissues

The Human Protein Atlas tissue database, which contains transcript and protein expression data [28] (https://www.proteinatlas.org/humanproteome/tissue) across all major organs and tissue types in the human body, was used to assign each network protein to a tissue expression class (“Not detected”, “Detected in single”, “Detected in some”, “Detected in many”, and “Detected in all”). This was done by downloading protein names provided in the pie chart (panel B) following this link https://www.proteinatlas.org/humanproteome/tissue/tissue+specific, by clicking on the respective area of the pie.

### Gene ontologies and subcellular localization

The SysGO database was used to obtain gene-specific ontologies and subcellular localization information [27]. The SysGO database contains (main) functional annotation for each protein-coding gene (321 classes; “SysGO—set 1”), of which 132 are related to signaling functions [27]. For visualization purposes, some classes are merged, resulting in a total of 58 groups (“SysGO—set 2”) [27]. This can be further reduced to 15 groups (“SysGO—set 3”) [27] that correspond to the classes of “Signaling”, “Metabolism”, “Protein translation, folding, modification and degradation”, “Transcription”, “Unknown”, “Cytoskeleton”, “Organelles”, “Other”, “Immune system and Inflammation”, “Chromatin organization and DNA repair”, “Neuronal System, synapses, channels”, “ECM organization”, “Cell junction and adhesion”, “Developmental”, and “DNA Replication”. Further, SysGO contains 47 subcellular localization groups (“SysGO localization—set 1”). For visualization purposes, these groups can be further merged into 19 classes, namely “Ribosomes”, “Cytosol”, “Nucleus” (merging of Nucleus, Nuclear envelope, Nucleoplasm, Chromatin, Nucleoli fibrillar center, Nuclear speckles, Nuclear bodies, and Necleoli), “Cell membrane”, “Microfilaments” (merging of Microfilaments (Actin), Intermediate filaments (Keratin, filaments), and Microtubules), “Mitochondria”, “Endoplasmic reticulum”, “Extracellular”, “Cell-ECM junctions”, “Golgi apparatus”, “Proteasome”, “Other vesicles” (merging of Melanosomes, Endosomes, Outer segments, and Lipid droplets), “Cilia, centrosome” (merging of Cilia, Centrosome, Microtubule organising centre, Midbody, and Mitotic spindle), “Cell–cell-junctions”, “Lysosomes”, “Peroxisomes”, “Focal adhesion sites”, “Cell cortex”, and “UNKNOWN”. The GO (gene ontology) functional enrichment was performed by running the PANTHER (protein annotation through evolutionary relationship; [29]) Overrepresentation Test (Released 20,210,224) for biological processes, by setting “GO biological process complete” as the annotation data set, “Homo Sapiens” as the reference list (20,595 genes), and by using a Fisher’s exact test at 95% level of confidence and false discovery rate correction (http://geneontology.org/). Following an analogous procedure, but with the annotation “PANTHER Pathways”, we obtained pathway enrichment scores for the network proteins downstream the Ras effector classes.

### Feedback loops

Our binary PPIs data is organized as an oriented graph (as defined in [30]) displaying *directed* interactions between pairs of proteins, clearly identifying the source and the target of every interaction. This formalism allowed us to determine the molecular feedbacks within our reconstructed network, by verifying the presence of a certain protein in the following downstream layers. In practice, we considered the signaling pathways downstream each L0 (Ras effector) and L1 protein, and searched the whole network to check if any downstream protein retroactively points to the given target (i.e. either L0 or L1 proteins), thus defining a feedback loop. Importantly, a feedback was defined as a backward regulation acting on a protein located at least 2 layers upstream (from L2 and L3 back to L0 proteins, and from L3 back to L1 proteins). This means that we excluded L1 to L0, or L2 to L1, interactions as these are not necessarily regulatory feedbacks, but may be e.g. scaffolding bounds or members of larger complexes.

### Literature analysis of SyGO processes linked to Ras-effectors

In order to find publications to validate the predicted SysGO process enrichments for the different effector classes, we used the search function of the PubMed database (https://pubmed.ncbi.nlm.nih.gov/). For each search, the name of the effector together with the process (sheet 1 of Additional file 10: Table S7) was used, and if publications were found, the content of the publication was reviewed and the pubmedID (PMID) was inserted (sheet 3 of Additional file 10: Table S7). If no publication was found, alternative protein names of effectors were inspected.

### Culturing of Caco-2 cells

Caco-2 cells (ATCC©HTB-37) were cultured in Dulbecco’s Modified Eagle’s Medium (Gibco, ThermoFisher Scientific, 21969-035) supplemented with 2 mM L-glutamine (Gibco, ThermoFisher Scientific, 25030-024), 10% (v/v) Foetal Bovine Serum (Gibco, ThermoFisher Scientific), and 1% Penicillin/streptomycin (Gibco, ThermoFisher Scientific), here called normal growth medium.

### Plasmids for exogenous expression of KRAS wildtype and mutant proteins

The four plasmids used in this work are based on the same backbone; pMDS_TetOn3G kozak-flag-GOI and differ only by their gene of interest (GOI) which are KRAS wildtype (WT), and KRAS mutant with the G12D, G12V, or G12C mutation (see also [31]). Plasmids were giftd by Hannah Benisty/ Luis Serrano (CRG Barcelona).

### Transfection, cell lysis and protein concentration

Caco-2 cells were seeded in 10-cm dishes in culture growth medium at day 0 and grown to 70–80% of confluency. Cells were transfected with 15 µg of plasmids (containing KRAS WT or KRAS G12D or KRAS G12V or KRAS G12C as gene of interest) using Lipofectamine 2000 (Invitrogen, 11668-019) according to the manufacturer’s instructions in OPTI-MEM reduced serum medium (Gibco, ThermoFisher Scientific, 31985-062) for 6 h. The medium was replaced by culture growth medium and for the KRAS WT supplemented with 50 ng/ml of doxycycline (Sigma-Aldrich). After 24 h cells were harvested, washed with phosphate buffer saline (PBS), and resuspended in 300 µl of lysis buffer [50 mM TRIS HCL pH 7.5, 1 mM EDTA, 1 mM EGTA, 150 mM NaCl, 2 mM MgCl2, 1 mM DTT, and 1% IGEPAL/NP-40 supplemented with PhosSTOP (Roche) and cOmplete, Mini protease inhibitor cocktail (Roche)]. Cells were lysed for 30 min on rotator at 4 °C and after centrifugation at 14000 rpm for 30 min at 4 °C, the supernatants were collected. Protein concentration was determined using the Pierce 660-nm Protein Assay (ThermoFisher Scientific).

### Affinity purification experiments in Caco-2 cells

Cell lysate from the Caco-2 cells transfected with the KRAS WT or MUTANT plasmids were precipitated from 800 µg of cell lysate using anti-Flag-M2 magnetic beads (Sigma, M8823) so that the immunoprecipitation protocol of the KingFisher DuoPrime purification system (ThermoFisher) can be used. Beads were washed in TBS (according to the manufacturer’s instructions) for 5 min, twice, at low speed. Then beads were collected by the KingFisher magnet and discarded into the samples wells and mixed at slow speed for 1 h. Then, beads-antibody-samples were collected and went through different wash salted solutions [Wash 1 and 2: RIPA buffer with 150 mM NaCl; Wash 3: RIPA buffer with 500 mM NaCl], mixed at low speed for 30 s. Then, beads-antibody-samples were eluted in 50 µl of glycine [0.1 M, pH 3.0] for 5 min. Immediately after, samples were neutralized with 20 µl of TRIS BASE (1 M, pH 8.0). To prepare for MS, samples were homogenized and denatured in urea (final concentration, 4 M), ammonium bicarbonate (100 mM), and calcium chloride (100 mM), then reduced in dithiothreitol (DTT) (final concentration, 1 mM) for 15 min and alkalinized in iodoacetamide (IAA) (3 mM) in the dark for 15 min. The next steps were carried out using the KingFisher; magnetic hydrophobic and hydrophilic beads (Sera-Mag SpeedBead Carboxylate-Modified Magnetic Particles) were added to the samples and mixed at low speed for 10 min, then beads-proteins were collected and washed in 80% of ethanol and released into the trypsin well (Promega, V5111) at a 50:1 (w/w) protein to protease ratio and mixed at low speed for 4 h of digestions at 37 °C. Beads were discarded and the resulting peptides were desalted, cleaned, and concentrated on C18Tips (ThermoFisher Scientific, 87784) [32] according to the manufacturer’s instructions, then resuspended in 0.15% trifluoroacetic acid and 2.5% acetic acid in mass spectrometry grade water.

### Mass spectrometry analysis

After trypsin digestion the samples were cleaned using C18 HyperSep SpinTips (Thermo Scientific), Samples were run on a Bruker timsTof Pro mass spectrometer (Bruker Daltonik) connected to a Bruker nanoElute nano-lc chromatography system (Bruker Daltonik). Tryptic peptides were resuspended in water with 0.1% (v/v) trifluoroacetic acid 0.1%. Sample was loaded on to a C18 trap (stainless steel trap cartridge, C18, 1 mm i.d. × 5 mm, (Part 160434, Thermo Fisher Scientific) at a approx. flow rate of 10ul/min with 100% buffer A (LC–MS grade water 99.9% and LC–MS grade acetonitrile with 0.1% (v/v) trifluoroacetic acid) Fisher Scientific (Thermo Scientific). Each sample was loaded onto a C18 analytical column Aurora UHPLC column (25 cm × 75 μm ID, C18, 1.6 μm) (Ionopticks). Separation was done in a linear gradient from 0 to 23 min buffer B increases from 5 to 32% at a flow rate of 300 nl/min, from 23 to 24 min buffer B increases from 32 to 95%, from 24 to 30 min the column is washed with 95% buffer B. The mass spectrometer, Bruker timsTof Pro was operated in positive ion mode with a capillary voltage of 1500 V, dry gas flow of 3 l/min and a dry temperature of 180 °C. All data was acquired with the instrument operating in trapped ion mobility spectrometry (TIMS) mode. Trapped ions were selected for ms/ms using parallel accumulation serial fragmentation (PASEF). A scan range of (300–1500 m/z) was performed at a rate of 10 PASEF MS/MS frames to 1 MS scan with a cycle time of 1.15 s.

The raw data was searched against the Homo sapiens subset of the Uniprot Swissprot database (reviewed, release-2020_02/) using the search engine Maxquant (version 1.6.17.0) using default parameters for trapped ion mobility spectra data dependent acquisition (TIMS DDA). Default search engine Maxquant were used with the following criteria: enzyme was set to trypsin/P with up to 2 missed cleavages. Carbamidomethylation (C) and oxidation (M)/acetylation (protein N-term) were selected as a fixed and variable modifications, respectively. Label-free quantification (LFQ) analysis was performed by employing the MaxLFQ algorithm as described (Cox et al., 2014). Peptide FDR 1%; Protein FDR 1%. “Label‐Free Quantitation; LFQ”, “iBAQ”, and “Match Between Run” settings were selected. No additional normalization steps were performed, as the resulting LFQ intensities are normalized by the MaxLFQ procedure [33]. The average number of peptides per protein was 8.6 and the average number of unique peptides per protein was 7.2. Protein IDs were matched to a unique gene name based on the SysGO database [27]. Six among the 43 effectors of the Ras-network were identified in the KRAS WT, G12D and G12V datasets (AFDN, ARAF, BRAF, RAF1, RIN1, RIN2) and four effectors were identified in the KRAS G12C AP-MS samples (AFDN, ARAF, RAF1, RIN1). Proteins identified in at least one of the KRAS AP-MS experiments (WT, G12D, G12V, G12C) were merged (1732 proteins) and used for comparison with proteins in the Ras network (Venn diagram in Fig. 7a).

### List of proteins identified by BioID in Caco-2 cells

A published dataset by Kovalski et al. [18] was used to obtain a list of proteins in complex with KRAS WT and KRAS G12D in Caco-2 cells. Data were obtained from Supplementary Table S1 “Mass Spectrometry Peptide-Spectrum Match Counts and SAINT Scores”, where all proteins with non-zero peptide intensities (based on columns F and G) were included. Protein IDs were matched to a unique gene name based on the SysGO database [27]. Three among the 43 effectors of the Ras-network were identified in the KRAS WT dataset (AFDN, RAF1, RASSF8) and nine effectors were identified in the KRAS G12D AP-MS samples (AFDN, RAF1, BRAF, RASSF8, RIN1, PIK3CA, ARAF, RAPGEF6, RASSF5). Proteins identified in at least one of the KRAS AP-MS experiments (WT, G12D) were merged (649 proteins) and used for comparison with proteins in the Ras network (Venn diagram in Fig. 7a).

### Protein complex databases

The Bioplex database [34] (https://bioplex.hms.harvard.edu/index.php) was used to obtain larger complexes of effectors obtained from multiple AP-MS experiments in HEK293 cells of individually flag-tagged Ras effectors. For 34 among the 43 effectors of the Ras network, AP-MS data were available in the BioPlex database (ARAF, BRAF, RAF1, AFDN, PIK3CA, PIK3CB, PIK3C2B, RIN1, RASSF1, GRB10, GRB14, SNX27, RASSF7, PIK3CD, PIK3C2A, PLCE1, RIN2, RIN3, TIAM1, ARAP1, DGKQ, RASSF2, RASSF3, RASSF6, RASSF8, RAPGEF2, RAPGEF4, RAPGEF6, APBB1IP, RAPH1, MYO9B, RGS12, RAPGEF5, RGL1). Protein IDs were matched to a unique gene name based on the SysGO database [27]. Proteins identified in at least one of the effector AP-MS experiments were merged (463 proteins) and used for comparison with proteins in the Ras network (Venn diagram in Fig. 7).

### CellDesigner software

CellDesigner 4.4.2 (http://www.celldesigner.org/) [35] was used to visualize the subnetwork of the 274 class-specific proteins and their interactors. The biochemical reactions have been distinguished between directed (single arrow) and undirected (double arrow), the proteins have been colored according to their effector class of belonging, and the various compartments permit to organize the proteins by their main subcellular localization.

## Results

### Reconstruction of a large-scale binary and layered Ras-effector signaling network

Ras proteins are pivotal to a large number of cellular processes, although the extent of Ras-mediated signaling pathways is still poorly understood. The present Ras-effector signaling network reconstruction challenges the widely known Ras-mediated MAPK and PI3K cascades, by integrating knowledge on direct interacting partners from various PPI databases. The output is an oriented graph of pairwise interactions defining a 3-layer signaling network downstream 43 Ras effectors, which belong to 12 functional classes (Additional file 1: Table S1).

In order to comprehensively analyze the Ras signaling network, we started from a set of 43 candidate Ras effectors (having a Ras binding domain) that are either well-established or predicted Ras binders [10] (see flowchart in the graphical abstract). From these, we built the downstream effector-mediated pathways, e.g., from RAF (Ras effector, i.e. layer 0 interactor) to MEK (layer 1 interactor) to ERK (layer 2 interactor) to MYC (layer 3 interactor), by searching for signaling molecules up to 3 layers downstream. Evidence for such molecular interactions was mainly retrieved from SignaLink [22] and KEGG Pathway/WikiPathways, and complemented with protein–protein interactions (PPIs) from STRING and HuRi (see Materials and methods). From SignaLink, we selected all the directed PPIs for *H. sapiens*, and then included any additional (directed) interaction from KEGG Pathway and WikiPathways (see Additional file 2: Figure S1). Yet, for 28 of the 43 effectors, this information is lacking. Therefore, we considered their first interacting partners from STRING and HuRi, that are *undirected* binary interactions. Eventually, only 2 effectors remained (ARHGAP20 and ARAP2) whose direct interactors are unknown.

**Fig. 1:**
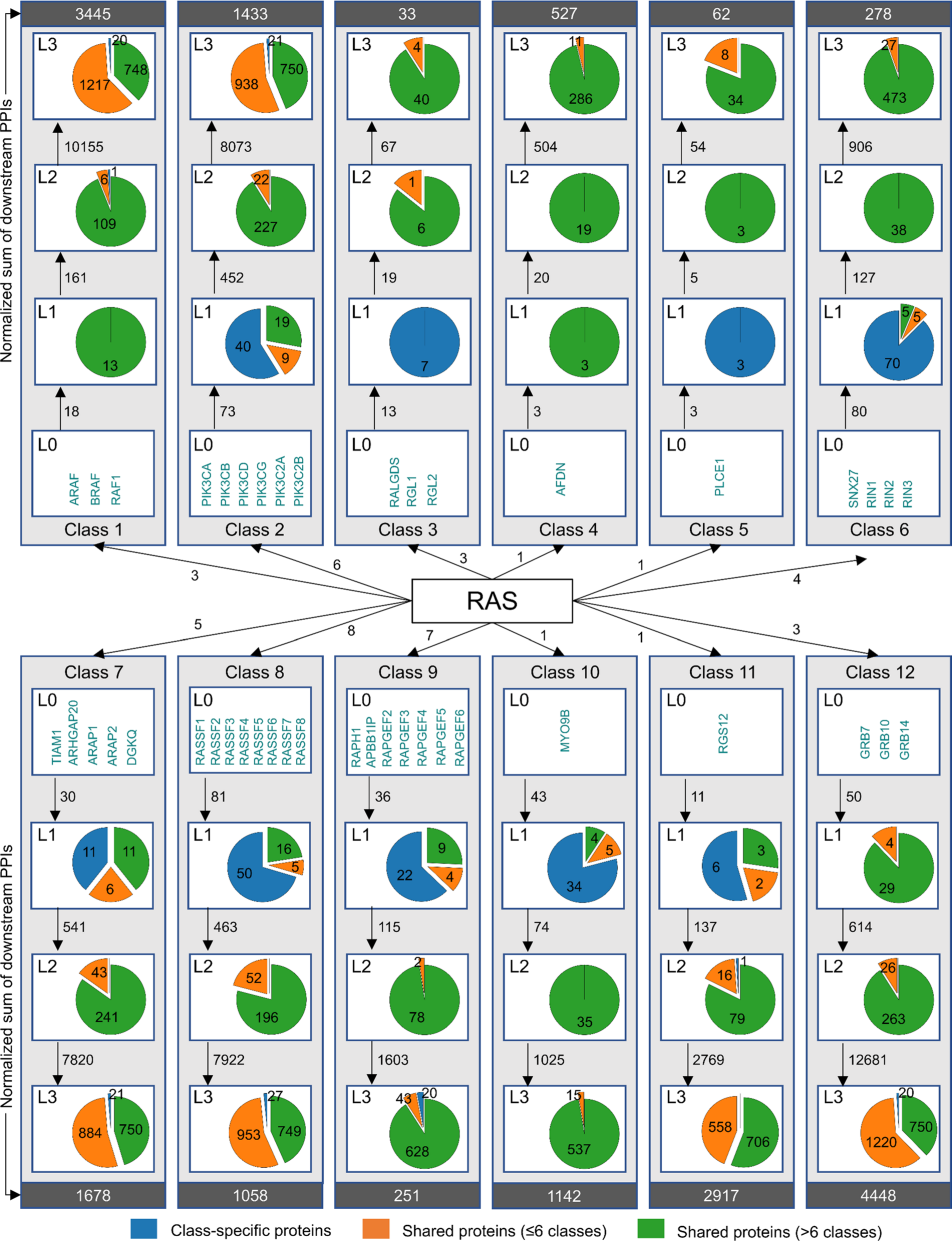
Overview of proteins and interactions of the Ras signaling network. Illustration of the 12 classes of Ras effectors and their downstream targets, categorized according to their cross-class presence (class specific, shared within 6 classes at most, or 7 classes at least). The numbers on the arrows indicate the numbers of interactions from one layer to the following. The normalized sum of downstream PPIs expresses the total PPIs per class divided by the number of effectors

We collected a total of 19,080 binary PPIs, increasingly distributed across the downstream layers: 441 PPIs in layer 1; 1660 in layer 2; and 16,979 in layer 3 (Fig. 1; flowchart in graphical abstract). In particular, this was obtained by first querying the Ras effectors for their direct interactors, defining the layer 1 (or L1) proteins of our signaling network. Those, in turn, have been used as a query to construct L2 proteins, selecting PPIs from SignaLink as explained above and in the Materials and methods section. Similarly, we also obtained L3 proteins. The resulting 2290 network proteins are to a large fraction (90%) expressed in many or all human tissues according to the Human Protein Atlas [28]. Thus, our network is likely applicable to multiple cell types and tissues.

The importance of a protein in a PPI network is often related to the number of interactions it is involved in. These highly connected proteins are called ‘hubs’ [36], and they often play central roles in a network as alteration in hub properties (e,g, mutations or abundance changes) have the potential to affect a large number of interacting partners. We calculated the number of interactions from the three layers of PPIs of the 2262 Ras-related downstream proteins (Additional file 1: Table S1) and we counted the number of occurrences of each protein of the network (both as a source and as a target) to explore the concept of protein centrality (Additional file 2: Figure S2; Additional file 3: Table S2). The Ras network centrality analysis allowed us to distinguish between hub and isolated proteins, based on the number of in-/outward interactions (in-/out-degree centrality; cf. Additional file 3: Table S2). Supplementary Figure S2 delineates the distinctive feature of signaling hubs and non-hubs, by showing that the majority of the proteins are non-hubs (having just a few interacting partners), and inversely, that only a few are hub proteins (having lots of interactors). Using a cut-off of at least 10 interactions to define a hub protein, the majority of proteins (65%) are non-hubs (Additional file 3: Table S2). Thus, our network adopted a typical scale-free network topology (following a power law) [37].

With the aim of characterizing and validating the obtained pairwise interactions that compose our signaling network, we also annotated each protein with its functional information and main subcellular localization, as per [27]. Furthermore, the 43 Ras effectors have been grouped into 12 classes to study the proteins downstream each class jointly. The resulting 2290 Ras-downstream proteins (including the effectors) were studied for their implication in the different classes (hence, for the signaling crosstalk), their subcellular localization, and their function.

### Crosstalk and feedback analysis

A total of 2290 signaling members have been identified (including the 43 Ras effectors), where most of them being shared among multiple signaling classes, and only 274 being class specific (Additional file 2: Figure S3; Additional file 4: Table S3). In order to investigate the degree of interconnectivity of the various signaling pathways modulated by Ras, we looked at the shared members of our network as a measure of the molecular crosstalk. Figure 1 illustrates the amount of shared and non-shared (i.e. class-specific) proteins downstream each of the 12 effector classes (L0) for every interaction layer (L1, L2, L3), as well as the number of interactions from one layer to the following (marked on the arrows). Remarkably, we observe a decrease in the amount of class-specific proteins with layer progression, indicative of crosstalk occurring especially downstream (layers 2 and 3). On the contrary, class specificity mostly takes action at the beginning (layer 1) of the signaling events. The number of downstream PPIs (normalized by the number of effectors by class) can vary widely across the classes, being highest in class 12 (RTK-Grb signaling), 1 (RAF-MEK-ERK signaling), and 11 (RGS-GPCR signaling), and lowest in class 3 (RalGEF-Ral-PLD-Sec5 signaling) and 5 (PLCε-DAG-IP_3_ signaling) (Fig. 1).

To gain new insights on the extensive crosstalk of such a network (i.e. to identify pathways that are likely to interact and hence influence each other), we analyzed the whole set of downstream proteins and the role they play in multiple pathways. In particular, we calculated how many proteins are found downstream one or many effector classes, considering all the possible combinations for the 12 classes, i.e. by singletons, pairs, triplets, etc. (e.g. classes 1, 2, 3, …; (1,2), (1,3), (1,4), …; (1,2,3), (1,2,4), …; and so on). We obtained 96 distinct (non-null) subgroups of classes among which the whole protein set is distributed, hence representing the extent of the network’s crosstalk (Additional file 2: Figure S3a and Additional file 4: Table S3). Additional file 2: Figure S3a illustrates how proteins populate each class-related subgroup. We observe that class 1 (RAF-MEK-ERK signaling) and 12 (RTK-Grb signaling) are often coupled, which is expected as they share 98.6% of the proteins downstream (cf. Additional file 2: Figure S4). Further, most of the proteins appear to be implicated in the following six signaling classes: RAF-MEK-ERK signaling (class 1), PI3K-AKT- signaling (class 2), RhoGEF-RAC-PAK signaling (class 7), RASSF-MST- Hippo signaling (class 8), RGS-GPCR signaling (class 11), and RTK-Grb signaling (class 12). This clearly illustrates the high level of crosstalk occurring within the network modulated by Ras, that interconnects and inter-regulates different signaling modules. Another aspect that is strictly related to such an extensive crosstalk is the regulation carried through feedback mechanisms. Indeed, our dataset revealed that 35% of the Ras effectors (i.e. AFDN, ARAF, BRAF, GRB10, GRB14, GRB7, PIK3C2B, PIK3CA, PIK3CB, PIK3CD, PIK3CG, RAF1, RASSF1, RASSF5, RIN1) appear at different layers downstream (e.g. RIN1 is a L0, L2, and L3 protein), suggesting that some feedback control might be in place (Additional file 4: Table S3). We further delved into this direction and explored the feedback loops downstream every (L0) effector and L1 protein (206 loops) (Additional file 2: Figure S3b; see also Materials and methods). By literature search, we confirmed that 27% of the proposed feedback cases are already known (Additional file 5: Table S4). Some well-documented examples include the RIN1-HRAS-loop (RIN1 is required for Rabex-5-dependent Ras ubiquitination; [38]), RAF1-PAK1-loop (PAK phosphorylates and activates RAF1; [39]), GRB7-ERBB2-loop (ERBB2 can phosphorylate and activate Grb7; [40]) and RASSF1-TP53-loop (TP53 binding to the RASSF1A promoter down-regulated RASSF1A expression; [41]).

### Subcellular localization

Every protein of our signaling network has been annotated with its main subcellular localization—e.g. cell membrane, nucleus, endoplasmic reticulum, etc. (Additional file 1: Table S1; Additional file 4: Table S3) following the Systemic Gene Ontology (SysGO) compilation in [27]. This enabled us to review the quality of our reconstructed network. On one hand, subcellular localization allowed us to assess whether a PPI occurs in “compatible” cell sections, according to our definition of PPI “localization compatibility” between pairs of compartments/organelles, e.g. cytosol and Golgi apparatus, or cell–cell junctions and (micro)filaments of actin and keratin, etc. (Additional file 2: Figure S5a). By applying this definition, we find that ~ 76% of our PPIs result compatible (Additional file 4: Table S3). As proteins often have multiple subcellular localizations (e.g. ERK kinase can be in the cytosol but is also translocated to the nucleus when activated [42]), we expect that the compatibility of 76% represents a lower limit and is potentially higher. Nevertheless, having the (main) subcellular localization accessible (Additional file 4: Table S3) is an additional benefit of our Ras network.

In a similar way, we used prior knowledge included by SignaLink under the column “Layer”. This latter comprises of six categories: “Interaction between pathway members” (e.g. scaffolds), “Directed protein–protein interaction” (e.g. binding of an adaptor protein to an active receptor), “Post-translational modification” (e.g. a kinase phosphorylating a substrate), “Transcriptional regulation” (e.g. a kinase phosphorylating a transcription factor), “Pathway regulation”, “Interaction from external databases”), the first four thereof we assumed to describe the case of two proteins directly interacting with each other (Additional file 2: Figure S5b). Notably, according to this second definition, based on the kind of interaction, as specified by SignaLink, we obtained ~ 80% of PPI compatibility, which is comparable to the portion attained with our “localization compatibility”. To note that the two compatibility definitions do not fully overlap, although they hold true for a comparable number of PPIs (20,767 localization-compatible interactions versus 21,838 layer-compatible interactions) and are in agreement in (2 + 57 =) 59% of the cases (Additional file 2: Figure S5c). Therefore, they represent two independent tools for the validation of our Ras signaling network, whose similar conclusions foster reliability in our approach.

**Fig. 2:**
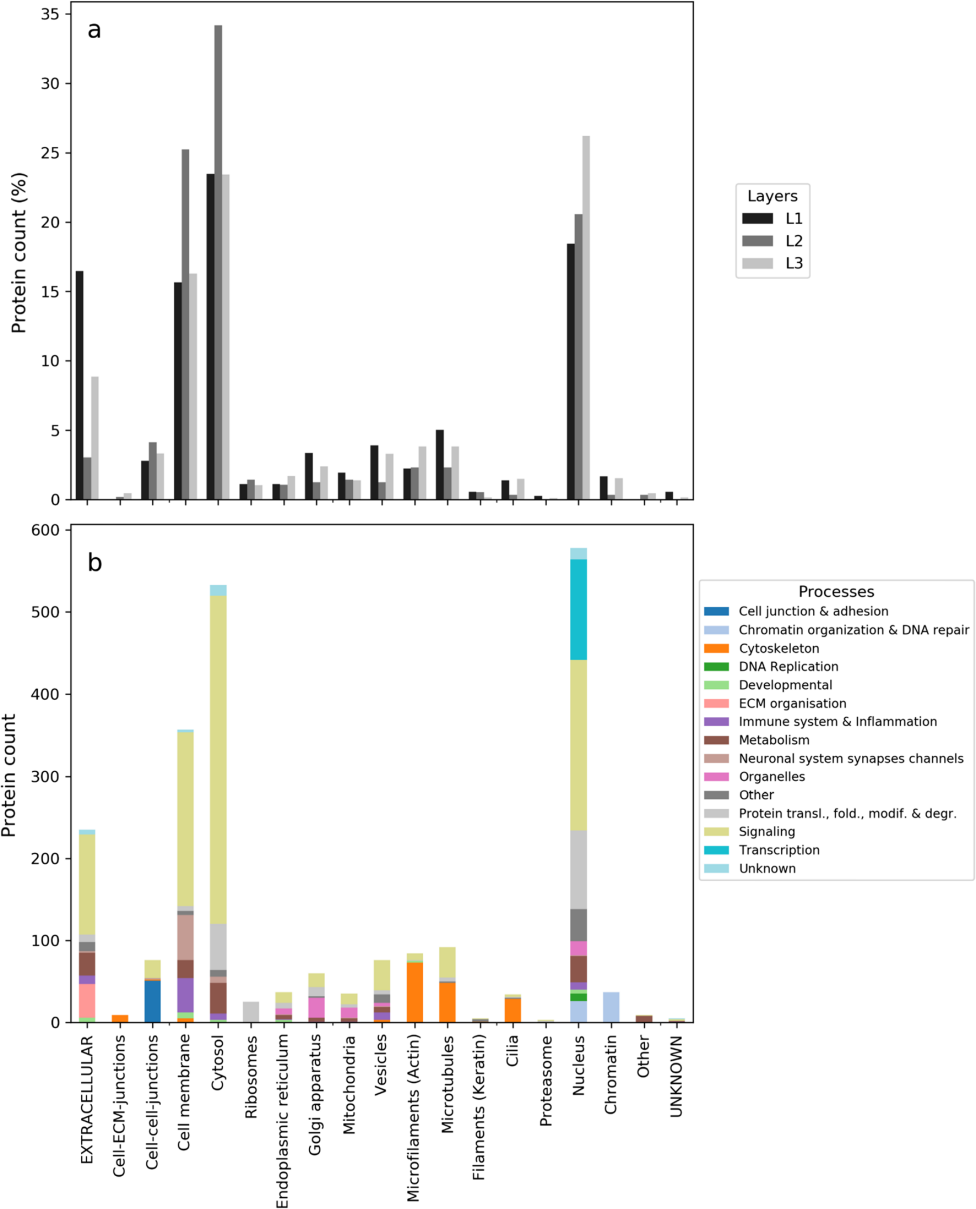
Analysis of the subcellular localization of proteins in the Ras signaling network. **a** Spatial subcellular localization of the downstream proteins by layer for the whole set of downstream proteins. **b** Analysis of the subcellular localization by protein function (SysGO Process (3))

Furthermore, we investigated the subcellular localization of all the proteins by layer (L1-2–3) in order to evaluate how ‘directional’ the downstream signal propagation is. In particular, we analyzed the relation between the effector interactors’ layer and localization. The results displayed in Fig. 2a confirm that, in the extracellular environment, there is a majority of L1 proteins, while in the cytosol, we observe mostly L2 proteins, and in the nucleus, primarily L3 proteins. However, at the cell membrane, the number of L1 proteins is especially exceeded by L2 proteins. This reinforces our assertion that feedback mechanisms are seemingly not rare at the first stages of the signaling events (involving Ras effectors themselves), and additionally we can reasonably assume that such feedbacks largely loop back to the membrane, hence explaining the L1-L2 numbers. Further, we examined the subcellular localization of our network proteins broken down by protein function based on 15 biological processes of SysGO [27] (Fig. 2b). We observe that the network proteins are mainly localized in the nucleus (with signaling and transcription functions), the cytosol (mainly signaling functions) and the cell membrane (mainly signaling functions). Other notable subcellular localizations are: extracellular environment (mainly signaling functions), microtubules (mainly cytoskeleton functions), and cell–cell junctions (mainly cell junction & adhesion functions).

### Functional analysis of network proteins

**Fig. 3:**
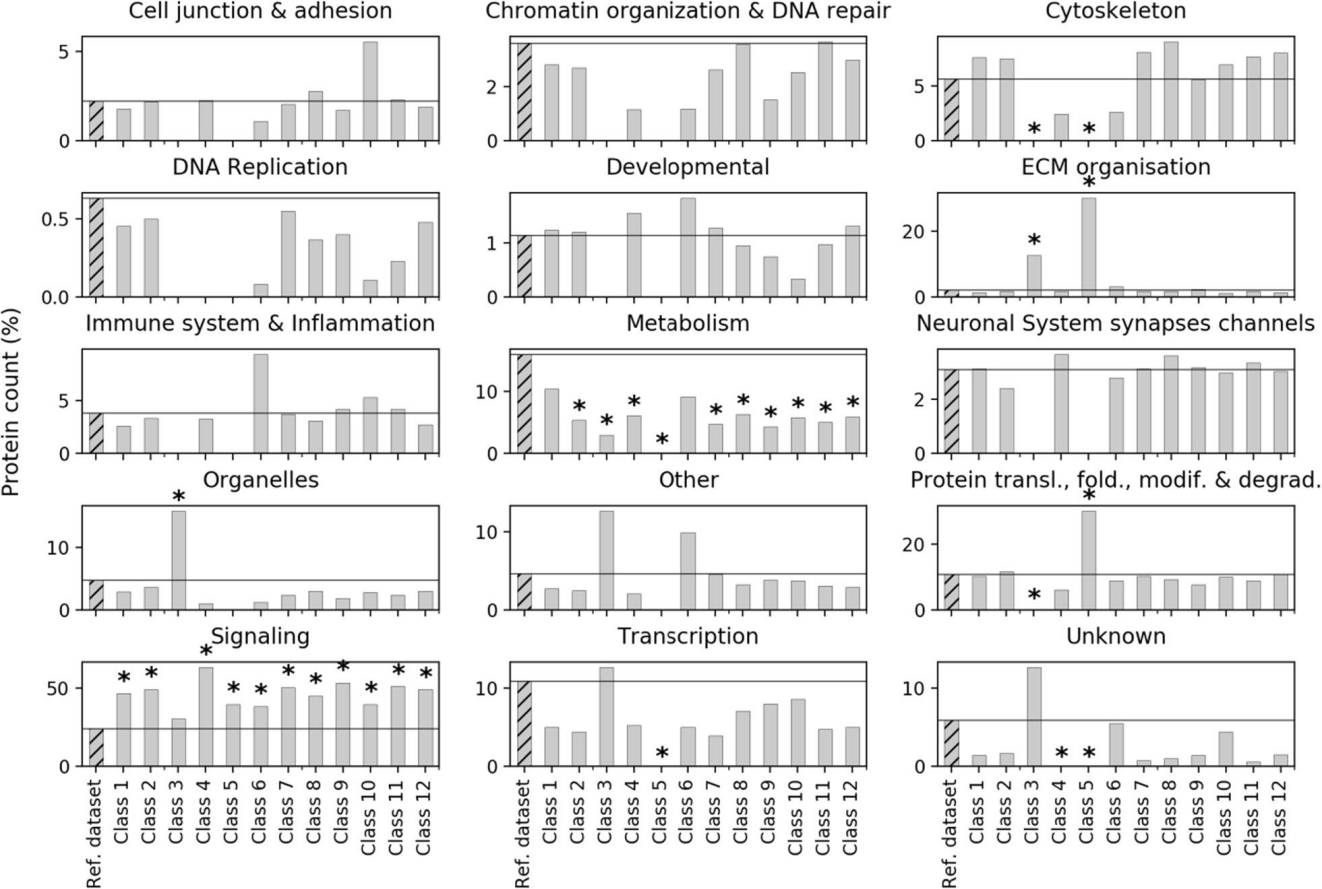
Functional analysis of the Ras signaling network according to 15 cellular processes. Functional enrichment with respect to a reference set of 19,300 proteins. The ‘*’ symbols indicate statistical significance at 0.05 of the respective Fisher’s exact test. The protein percentages were obtained for each class by summing up the proteins after dividing by the number of effector classes each protein is involved in and normalizing by the class-specific sum, as explained in the main text

The SysGO database [27] was used to characterize functional enrichments among the proteins downstream of the 43 effectors and 12 effector classes. To account for protein share across the different classes and avoid over-representation due to cross-signaling, we normalized each protein by the number of classes it is involved into (e.g. AAAS participates to class 1 and 12, hence is assumed to be equally distributed between the two for an amount of 0.5; Additional file 6: Table S5). The functional analysis of such normalized proteins has been performed according to 15 SysGO (level 3) processes such as transcription, metabolism, signaling, etc.. Notably, we observe that the Ras-related network is highly specialized in signaling processes, as those constitute the 30–60% of the whole functional classification. We thus performed an enrichment analysis against a reference set of 19,300 proteins that have been previously characterized, especially by their function and subcellular localization, in [27]. Figure 3 depicts the functional enrichment broken down by class, in comparison with the reference set (rounding was applied to deal with integer values; Additional file 6: Table S5). In particular, we show evidence for nearly all effector classes to be significantly enriched in signaling-associated functions and depleted in metabolic processes (Fig. 3). Moreover, class 3 (RalGEF-Ral-PLD-Sec5 signaling) function turned out to be highly specialized towards organelles and extracellular matrix (ECM) organization, while class 5 (PLCε-DAG-IP_3_ signaling) is rather involved in ECM organization and protein translation, folding, modification and degradation (Fig. 3).

**Fig. 4:**
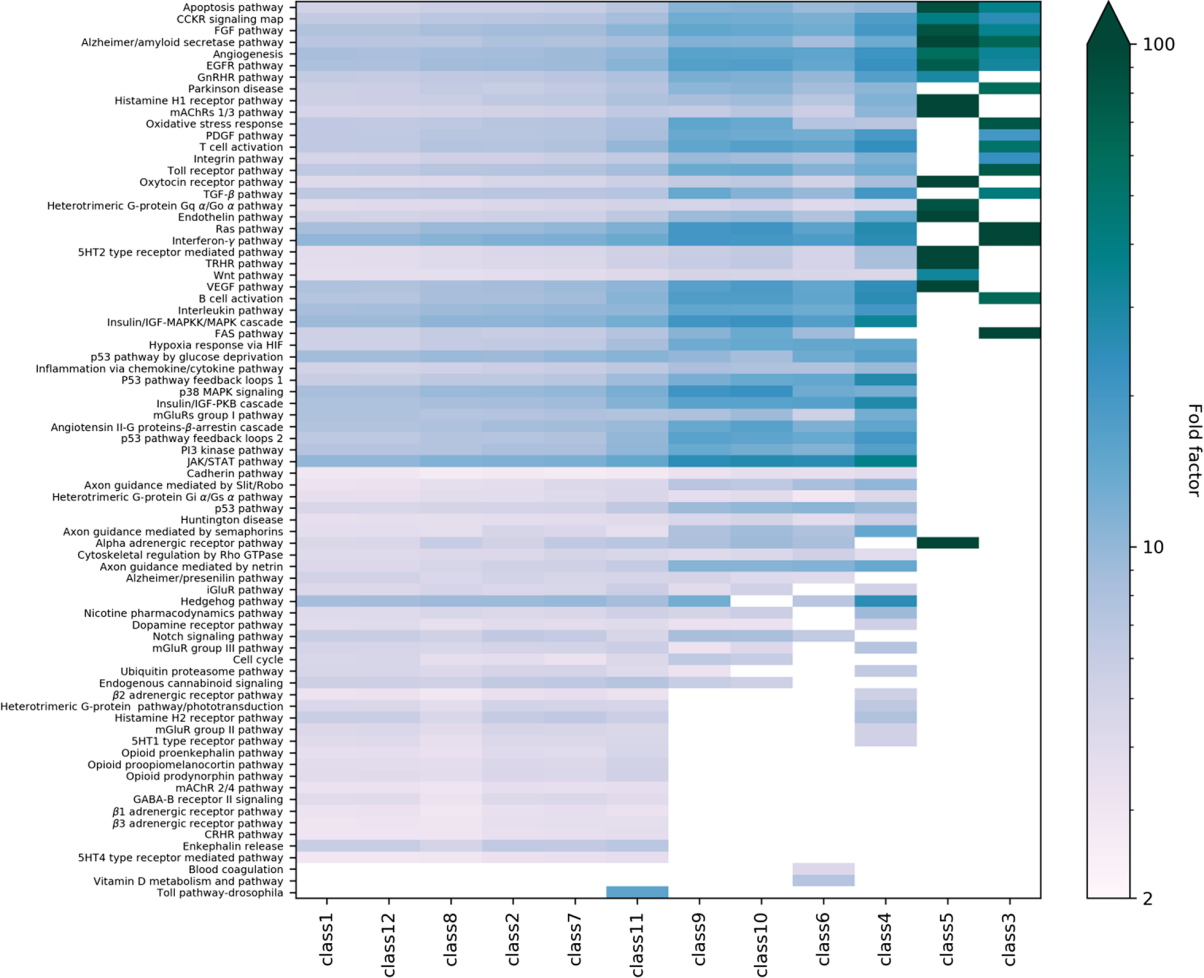
Pathway analysis by class. The threshold for enrichment is set at twofold. The 12 effector classes are ordered according to the clustering outcome of a principal component analysis (Additional file 2: Figure S6)

Furthermore, we performed an enrichment analysis of the Ras-downstream pathways (by class), using PANTHER [43] (Additional file 7: Table S6). Figure 4 compares the fold enrichment for the different pathways and classes (with classes ordered by PCA similarity; see Additional file 2: Figure S6). Interestingly, RAF-MEK-ERK signaling (class 1), RTK-Grb signaling (class 12), RASSF-MST-Hippo signaling (class 8), PI3K-AKT signaling (class 2), RhoGEF-RAC-PAK signaling (class 7), and RGS-GPCR signaling (class 11) cover many different pathways; while the remaining classes are associated to more specific pathways, especially PLCe-DAG-IP_3_ signaling (class 5) and RalGEF-Ral-PLD-Sec5 signaling (class 3). As expected, class 1 (RAF-MEK-ERK signaling) and 12 (RTK-Grb signaling), that share 98.6% of the proteins downstream (Additional file 2: Figure S4)—as already highlighted by the amount of proteins shared across classes (Additional file 2: Figure S3)—have a very close profile.

### Class-specific subnetwork and insights into class-specific functions

**Fig. 5:**
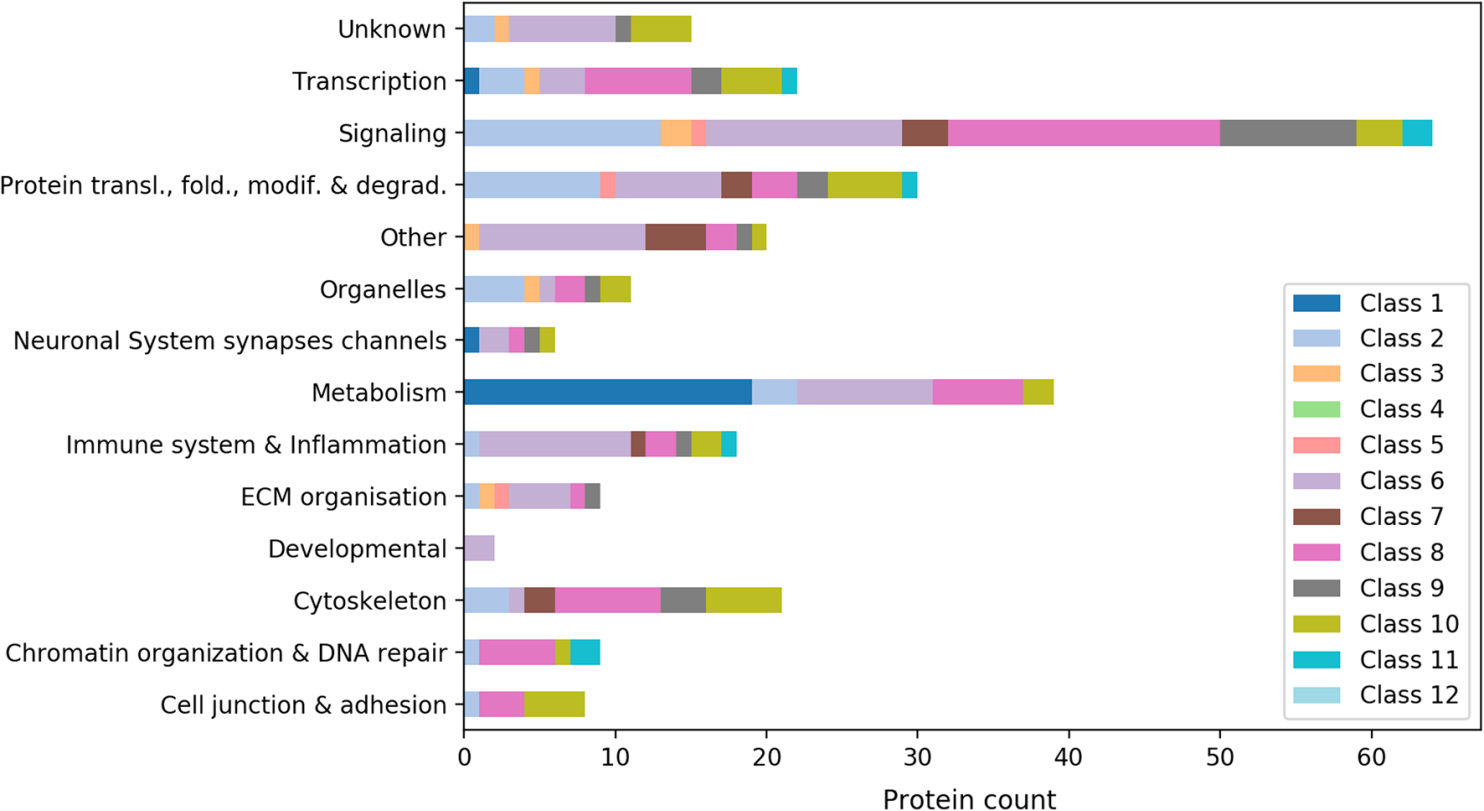
Functional analysis of the 274 class-specific (non-shared) proteins by process and class

With the aim of investigating class specificity, we undertook two approaches to characterize each particular effector class. The first approach delves into the processes related to the non-shared (class-specific) proteins of our Ras network, and the second one into the processes enriched in exclusively one effector class. We considered the subnetwork of the 274 class-specific proteins and examined them according to their functional annotation (Fig. 5). In particular, we observe that overall signaling-related activities dominate. This is also true for each effector class considered independently, except for RAF-MEK-ERK signaling (class 1) being more implied in metabolic activities, the RhoGEF-RAC-PAK signaling (class 7) involved in cytoskeleton reorganization, and Myosin-Actin signaling (class 10) having a major role in protein lifecycle, transcription, and cell junction and adhesion. Interestingly to note, none of our class-specific proteins is specifically involved in DNA replication. A visual representation of such a subnetwork (created with CellDesigner) is available as both xml file and pdf file (Additional file 8: Network S1; Additional file 9: Network S2). It displays the class-specific proteins (color coded by effector class) and their interactors within a compartmentalized virtual cell environment.

Following our second approach, we performed a complete GO biological process analysis, based on PANTHER overrepresentation test, to examine the enriched processes that characterize uniquely one class. By matching PANTHER GO terms with SysGO Process classification (set 1 and 3 [27]), we could assign those to the following macro-categories: cell junction and adhesion, chromatin organization, cytoskeleton, development, extracellular matrix organization, immune system and inflammation, metabolism, neuronal system, organelles, protein lifecycle, signaling, transcription, and other processes (Additional file 10: Table S7). To note that the PANTHER to SysGO Process (1) mapping gives some duplicate entries, in which cases the fold enrichments were averaged.

**Fig. 6:**
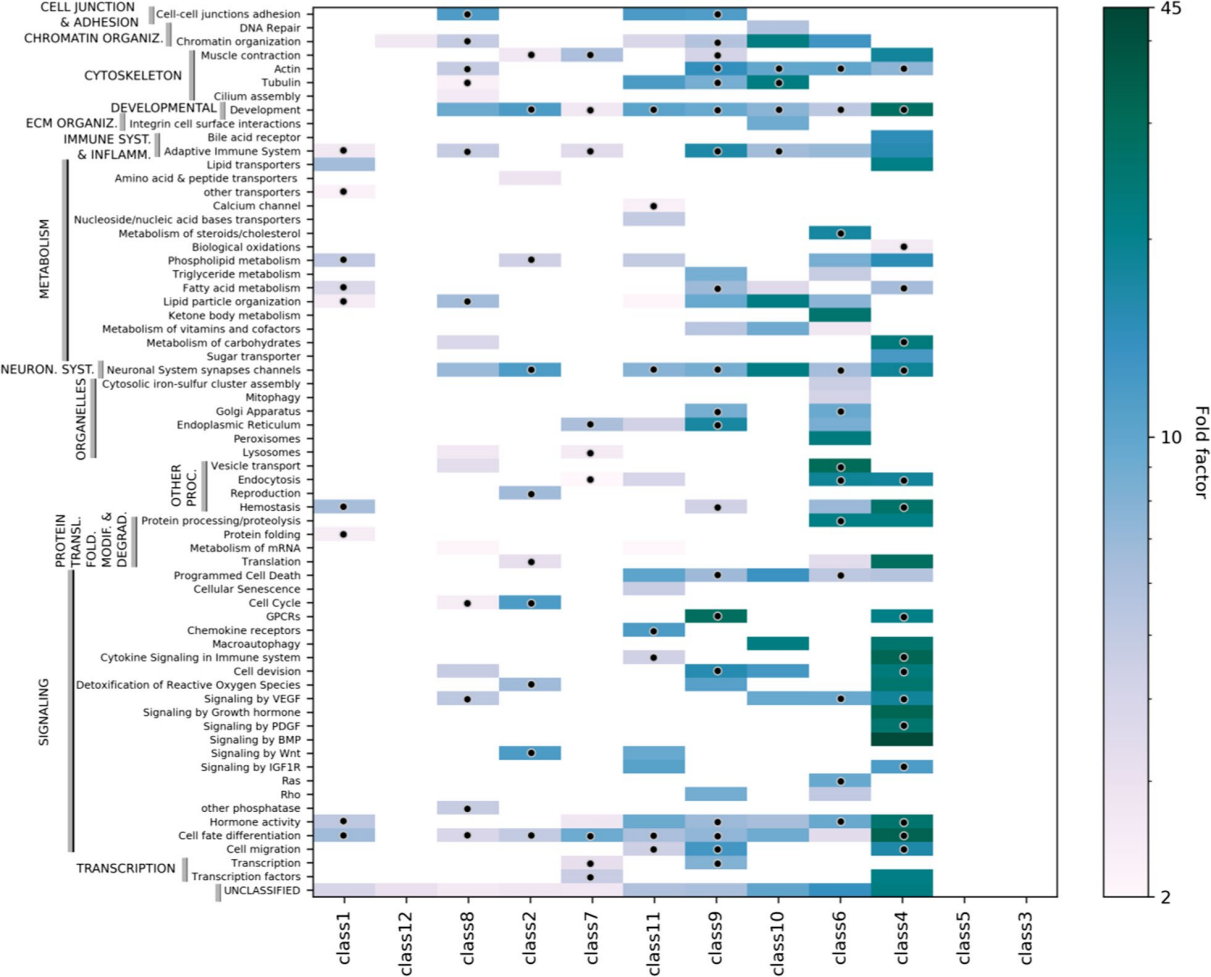
Biological process analysis. The threshold for enrichment is set at twofold. The 12 effector classes are ordered according to the clustering outcome of a principal component analysis (cf. Additional file 2: Figure S6). The dots indicate whenever a given effector-related process has been previously reported in the literature (Additional file 10: Table S7)

Figure 6 represents the class-specific enriched processes (fold change > 2) and provides a key to understanding how e.g., the diverse signaling functions, can be differentially dispatched throughout the 12 effector classes, each one being specialized in a different set of processes. Furthermore, for such a class-specific subnetwork, we observed a differential process enrichment that is especially remarkable for the SysGO Processes (1) relating to signaling and metabolic activities (Additional file 10: Table S7 and Additional file 2: Figure S7), which is in agreement with the enrichment analysis on the whole dataset (Fig. 3) indicating signaling and metabolic functions as the most significantly different from the reference dataset. Importantly, 58% of the class-process relations that we show here, have been previously reported in the literature (cf. dots in Fig. 6 and see Additional file 2: Figure S8). Such literature reports are detailed in Additional file 10: Table S7 and refer to antecedent studies having described the association between various *Ras effectors* and the biological processes that we found enriched. In this regard, it is worth mentioning that the process enrichment analysis does not depend on the Ras effector directly, but rather on the quality of the reconstructed signaling network *downstream the effectors*, which grants further confidence in our approach. In particular, we observe a remarkably high agreement between published data and our findings concerning the following signaling classes (Additional file 2: Figure S8): RAF-MEK-ERK signaling (class 1; 90% of processes confirmed), RhoGEF-RAC-PAK signaling (class 7; 90%), PI3K-AKT signaling (class 2; 85.7%), and RapGEF-RAP signaling (class 9; 79.2%). Therefore, we anticipate that some of the predicted (enriched) processes that are new to this study will find confirmation in future experimental research.

### Comparison of the Ras-effector signaling network with larger Ras- and effector-mediated complexes

Our Ras-effector signaling network has been issued from the integration of several sources and represents a dataset comprehensive of different experimental settings, cell types and conditions, which result in an interaction network that may be considerably broader (and less specific) than the one that can be empirically observed. Indeed, a comparison with experimental measurements is helpful to assess the capability of our reconstructed network to interpret data from larger protein complexes, e.g. proteins detected by affinity purification-mass spectrometry (AP-MS) [44] or proximity-dependent biotin identification (BioID) [45].

**Fig. 7:**
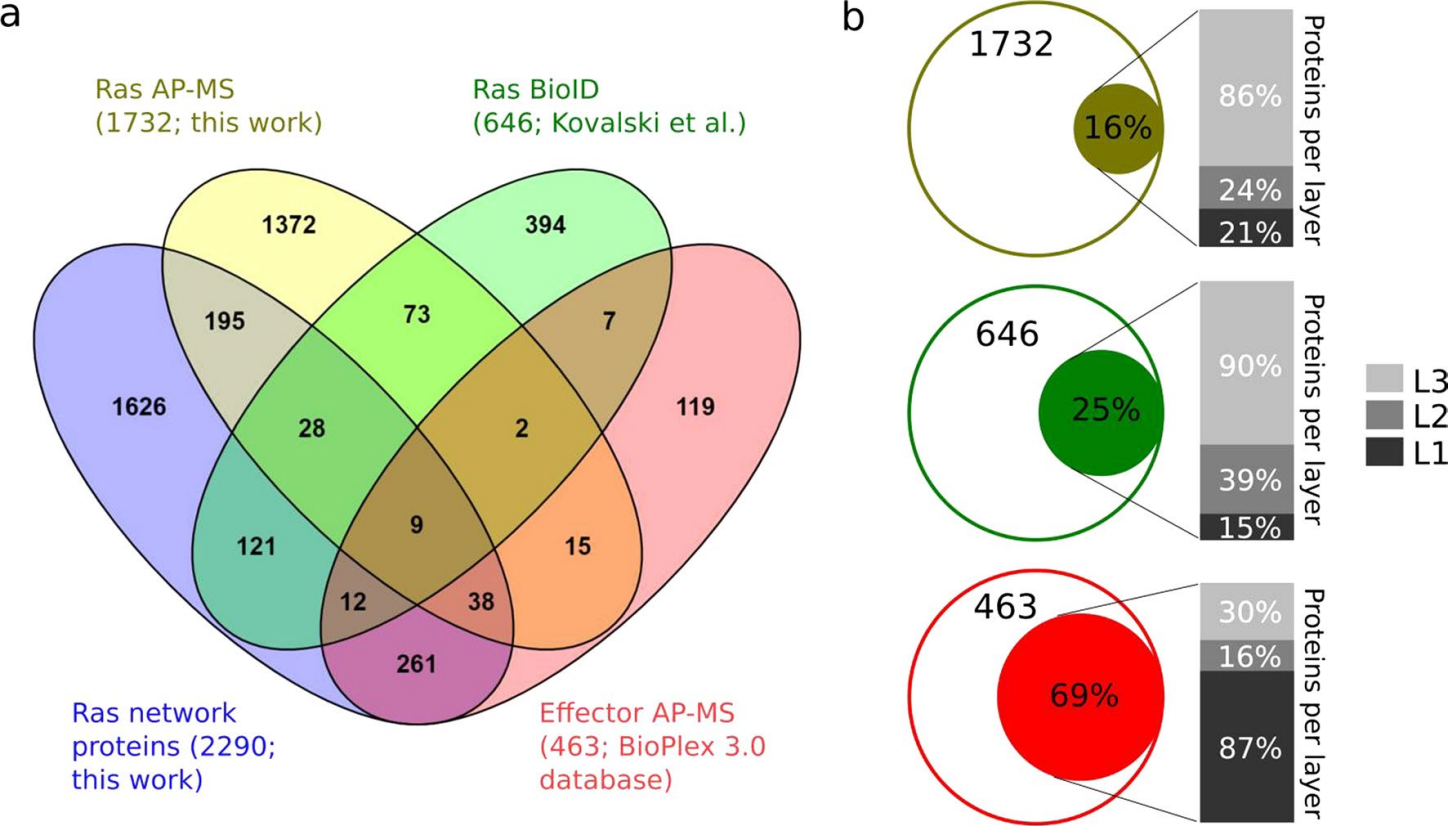
Coverage comparison between the Ras signaling network reconstructed in this work and 3 experimental network sources. **a** Venn diagram of the protein overlap with the following data: our in-house AP-MS measurements of KRAS-Flag-Tag in Caco2 cells performed by Camille Ternet, proximity ligation assay (BioID) on KRAS in Caco2 cells (Kovalski et al., 2019), and effector-specific AP-MS data from BioPlex database. The total number of proteins is indicated in the parentheses, for each dataset. **b** Portion of proteins, from each experimental dataset, in common with our reconstructed network (percentages within the sets), and their distribution by layer (stacked bar plots). The proteins belonging to multiple layers are counted the number of times they appear, explaining why the sum of the stacked bar plots exceeds 100%

We thus calculated the overlap between the 2290 Ras network proteins and AP-MS/BioID datasets of Ras and effectors (Fig. 7a). The first dataset contains in-house AP-MS measurements performed on Caco2 cell lines transfected with Flag-tag KRAS wildtype or oncogenic mutants (G12D, G12V and G12C). In total, 1732 proteins were identified in the union of proteins identified in at least one of these AP-MS experiments, of which 6 were effector proteins. The second dataset concerns KRAS (WT or G12D) BioID proteomics experiments, also in Caco2 cells [18]. The union of all proteins identified in at least one experiment was 649 proteins, including 9 effectors. The third dataset regards effector-specific AP-MS data obtained from the BioPlex database [34]. AP-MS experiments for 34 effectors were available in the BioPlex database and the union of all proteins identified in at least one Effector AP-MS was 463. The portion of proteins experimentally detected in KRAS AP-MS, BioID and effector AP-MS, that are also found in our assembled Ras network, is respectively 16%, 25%, and 69% (Fig. 7b; Additional file 10: Table S8). In particular, these first two percentages primarily comprise of proteins from layer 3 (86% and 90%, respectively), which are further downstream; whereas, the last percentage is mainly composed by layer 1 proteins (84%), which are the most proximal to the effector layer. Intriguingly, in the BioPlex experiments, we also observe the highest presence of L1 proteins, which we think may be due to the nature of the experimental technique itself, or perhaps to data post-treatment aiming at clearing away “contaminants” and interactions with a low certainty score, which might have resulted in the removal of many of the interactions occurring further downstream in L2 and L3.

Once again, we like to stress that the constructed Ras-effector network is a generic network, which has been constructed from interaction information from a large variety of experiments in different cells and contexts. Thus, it is not expected that one AP-MS or BioID experiment in a specific cell line will capture all interactions of our Ras network. Likewise, it is not expected that the constructed Ras-effector network is exhaustive, as information in interaction databases is also not complete and, in particular, many cell-type specific interactions are likely lacking. Overall, the experimental overlap with our collected PPIs—especially for the effector AP-MS data,—is encouraging and corroborates our Ras network’s utility and applicability.

## Discussion

Ras proteins are key signaling hubs that are activated by a number of cellular receptors and control cellular phenotypes via signaling networks downstream of effector proteins. Understanding these networks in different physiological conditions and how they are rewired in the context of diseases such as cancer [2] is critical in order to apply mechanism- and network-centric approaches in precision oncology [46, 47]. Indeed, pathway circuitry and rewiring is a keystone concept in network-based approaches to disease.

Here, we challenged the ‘old’ framework and looked outside the canonical Ras effector pathways. Our Ras-effector network comprises 43 effector proteins that converge onto 12 effector classes. Some of these effectors have only weak binding affinities between their RBDs and the active Ras (i.e. RasGTP), but these may be binding to RasGTP in significant amount if additional domains present in these effectors are recruited to the plasma membrane [10]. With 2290 proteins connected in 19,080 interactions, this represents, to the best of our knowledge, the largest representation of a network downstream of Ras proteins to date. Importantly, the network is represented as an oriented graph of direct pairwise interactions that form 3 layers downstream of each effector, integrating data from signaling pathways and protein–protein interaction databases. The main purpose of our Ras-effector network was to generate an up-to-date literature network that includes all effectors that have the potential to be in complex with Ras in specific cell types, tissues or contexts (microenvironmental stimuli). It should set the framework for the analysis and expansion with additional (more cell type- and condition-specific) experimental network data in the future.

The quality of our Ras-effector network was assessed in three ways. Firstly, we used subcellular localization information to show that ~ 76% of the PPIs occur in compatible compartments. This might even be the lower bound of compatibility since SysGO only takes the most prevalent, and not secondary, localizations into account. Secondly, we evaluated the biological processes enriched in our Ras-effector network and determined that, on average, 58% found confirmation in the literature (the agreement is higher for well-studied classes such as RAF-MEK-ERK signaling, RhoGEF-RAC-PAK signaling, and PI3K-AKT signaling). Lastly, we showed a good overlap between Ras-effector network proteins and proteins belonging to larger complexes as identified by AP-MS and BioID experiments on Ras and effector proteins (despite only few effectors present in those AP-MS experiments). In particular there is a high overlap (61%) between BioPlex effector complexes and our network proteins, suggesting that in the future our network can be used to further break down larger complexes obtained by AP-MS experiments into smaller sub-complexes. However, there are still 1618 proteins in our Ras-effector network that have not been detected in any of the AP-MS or BioID experiments analyzed here. As those experiments were performed in specific cell lines (Caco2 cells for the in-house AP-MS and BioID data, and HEK293 cells for the effector AP-MS), it is expected that a different subset of our general Ras-effector network will be confirmed in different contexts or disease-/drug-related conditions (e.g. [17]). While a high proportion of our Ras-effector network proteins are likely to be expressed in most cells and tissues according to the Protein Atlas database [28], the use of different cell lines and, in particular, performing Ras AP-MS experiments in different patho-/physiological conditions (e.g. various ligands and growth factors, hypoxia, inflammation, etc.) could nevertheless result in a yet higher overlap of network proteins. For example, we have recently predicted, using mathematical modeling, that a high fraction of effector proteins only efficiently binds to Ras only in specific conditions, e.g. when effectors are additionally recruited to the plasma membrane via other than their RBD to Ras-GTP [10]. Indeed, Ras AP-MS experiments obtained in Caco2 cells for different stimuli/growth conditions show that the total number of effectors and other proteins in the Ras-mediated complex increases with the number of conditions (C. Ternet, unpublished data).

A functional analysis of the 12 effector pathways and associated proteins downstream of Ras shows enrichment in signaling-related functions and depletion in metabolism. Other functions associated to proteins specific to some effector classes of our network are organelles, cytoskeleton, extracellular matrix, and protein folding, modification and degradation. We also generated a sub-network that includes only class-specific proteins, which was represented as a CellDesigner diagram. Interestingly, while in our complete Ras-effector network, metabolic proteins were underrepresented, the class-specific sub-network shows a particularly high number of metabolic enzymes in class 1 effectors (RAF-MEK-ERK signaling).

A high number of crosstalk was identified in the Ras-effector network, which is indeed a key property of signaling pathways [48–50]. As a proof of evidence of this feature, the database XTalkDB [51] (http://www.xtalkdb.org) was recently developed to document crosstalk between various signaling pathways. Here, we chose to focus on 206 crosstalk network structures involving the effector proteins, that are likely to be implicated in regulatory feedback loops. Indeed, 27% of these structures have been already described as feedbacks in previous independent studies, and we confirmed some well-known examples such as the retroaction from active PAK, or ERK kinases, to RAF1 [39, 52]. Further investigation of our predicted crosstalk and feedback mechanisms is needed. This will be valuable to shed some light on complex systems behaviors that are dependent on both cellular context and molecular competition, which can drive different outcomes and phenotypes, and achieve signal specificity [15, 48, 53]. These features, together with multi-omics data integration (e.g. proteome differential expression), can have an impact on drug activity and implications in targeted therapies [54, 55].

## Conclusions

Our large-scale reconstruction efforts for the Ras-effector signaling network will call for context-/tissue-/cell type-/condition-specific refinements from experimental recognition and validation. This will be a crucial steppingstone to redefine the network boundary conditions in view of formal topological pathway analyses (e.g. [15]) that will elucidate systems properties (e.g. input–output relationships, crosstalk, pathway redundancy, etc.), guide mathematical modeling, and allow mechanistic investigation. Considerable research has been undertaken in favor of disrupting activated Ras-effector protein complexes as a therapeutic strategy acting at the level of signaling interactions, and promising evidence has been increasingly reported [7, 56]. Such advancements go hand in hand with the objective to narrow down the Ras network to make it (patho-)physiologically useful, unraveling the mechanisms that make signal transduction selective and specific in different cells.

## Supplementary Information

**Additional file 1: Table S1.** All protein-protein interactions (PPI) of the Ras-network sorted by layer.

**Additional file 2: Figure S1.** Coverage of the Ras network with directed vs undirected interactions. **Figure S2.**. Network centrality analysis for hub and non-hub proteins. **Figure S3.** Crosstalk of the Ras-effector downstream proteins across the 12 effector classes. **Figure S4.** Shared proteins among the 12 effector classes. **Figure S5.** Subcellular localization and interaction compatibility. **Figure S6.** PANTHER GO pathways analysis of the 12 effector classes. **Figure S7.** Differential enrichment of the SysGO Processes (1) by effector class. **Figure S8.** Comparison of the enriched class-specific processes with literature reports.

**Additional file 3: Table S2.** Hub protein analysis.

**Additional file 4: Table S3.** Downstream proteins of the Ras network.

**Additional file 5: Table S4.** Effector-related feedback analysis.

**Additional file 6: Table S5.** Functional analysis of the Ras network proteins using SysGO.

**Additional file 7: Table S6.** Pathway enrichment analysis using PANTHER.

**Additional file 8: Network S1.** CellDesigner diagram as zip.

**Additional file 9: Network S2.** CellDesigner diagram as pdf.

**Additional file 10: Table S7.** Biological process enrichment of class-specific proteins.

**Additional file 11: Table S8.** Coverage comparison between the Ras signaling network reconstructed in this work and 3 experimental network sources.

## Data Availability

All data generated or analyzed during this study are included in this published article and its supplementary information files.

## Abbreviations

AP-MS: Affinity purification combined with mass spectrometry
BioID: Proximity-dependent biotinylation identification
GO: Gene ontology
GOI: Gene of interest
MAPK: Mitogen-activated protein kinase
PI3K: Phosphatidylinositol 3-kinase
RBD: Ras binding domain
PPI: Protein–protein interaction
