# Supplementary material for: Reconstruction and analysis of a large-scale binary Ras-effector signaling network
Source: Cell Commun Signal. 2022 Mar 4;20:24. doi: 10.1186/s12964-022-00823-5 (PMC8896392; doi:10.1186/s12964-022-00823-5)
Supplement: Supplementary file 3 — Additional file 2: Figure S1. Coverage of the Ras network with directed vs undirected interactions. Figure S2.. Network centrality analysis for hub and non-hub proteins. Figure S3. Crosstalk of the Ras-effector downstream proteins across the 12 effector classes. Figure S4. Shared proteins among the 12 effector classes. Figure S5. Subcellular localization and interaction compatibility. Figure S6. PANTHER GO pathways analysis of the 12 effector classes. Figure S7. Differential enrichment of the SysGO Processes (1) by effector class. Figure S8. Comparison of the enriched class-specific processes with literature reports. [file 12964_2022_823_MOESM3_ESM.pptx]

## Slide 1
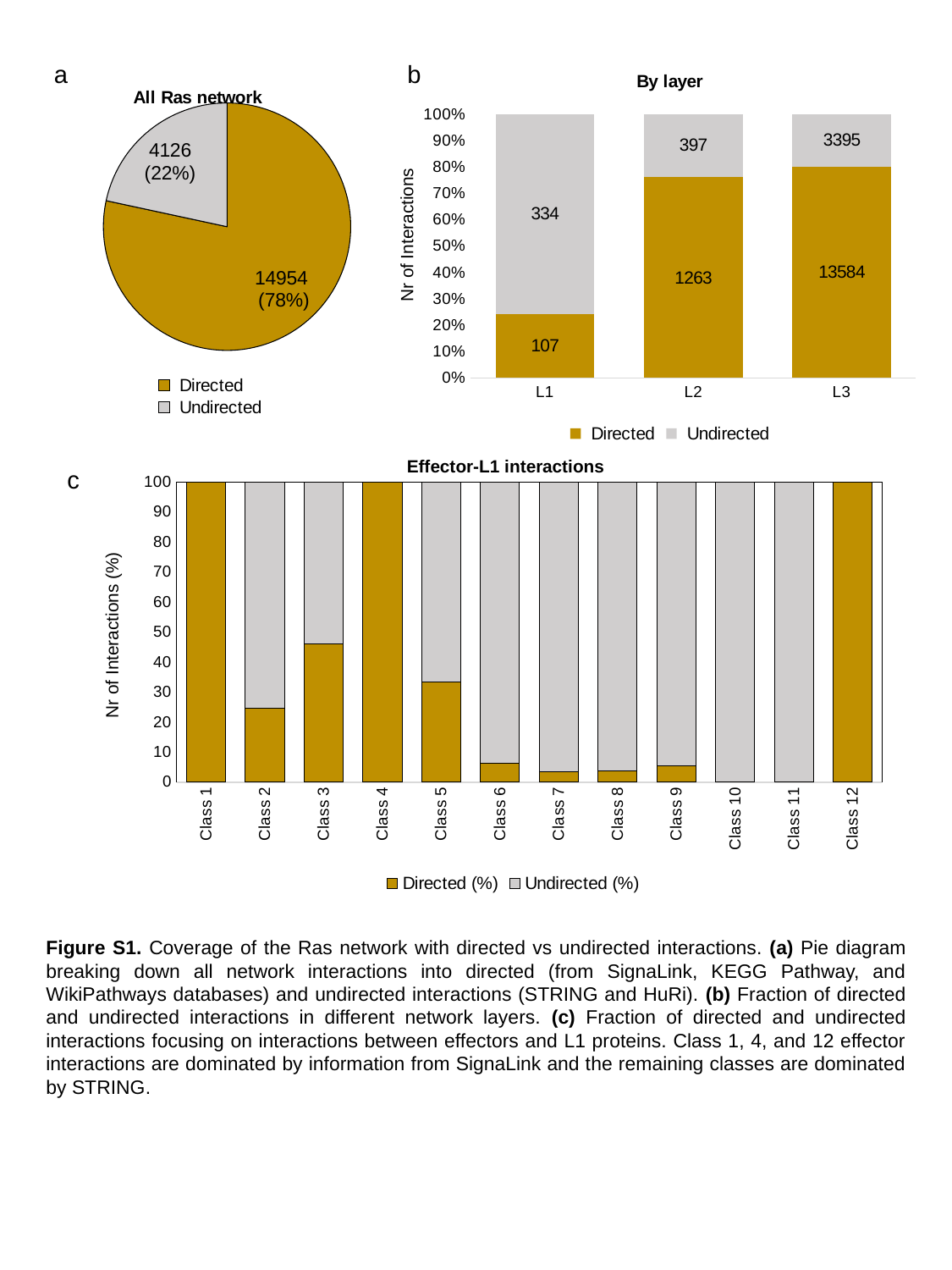

### Chart: All Ras network
| Category | |
|---|---|
| Directed | 14954.0 |
| Undirected | 4126.0 |
### Chart: By layer
| Category | Directed | Undirected |
|---|---|---|
| L1 | 107.0 | 334.0 |
| L2 | 1263.0 | 397.0 |
| L3 | 13584.0 | 3395.0 |a
b
Nr of Interactions
Effector-L1 interactions
c
### Chart
| Category | Directed (%) | Undirected (%) |
|---|---|---|
| Class 1 | 100.0 | 0.0 |
| Class 2 | 24.65753424657534 | 75.34246575342466 |
| Class 3 | 46.15384615384615 | 53.84615384615385 |
| Class 4 | 100.0 | 0.0 |
| Class 5 | 33.33333333333333 | 66.66666666666666 |
| Class 6 | 6.25 | 93.75 |
| Class 7 | 3.3333333333333335 | 96.66666666666667 |
| Class 8 | 3.7037037037037033 | 96.29629629629629 |
| Class 9 | 5.555555555555555 | 94.44444444444444 |
| Class 10 | 0.0 | 100.0 |
| Class 11 | 0.0 | 100.0 |
| Class 12 | 100.0 | 0.0 |Nr of Interactions (%)
Figure S1. Coverage of the Ras network with directed vs undirected interactions. (a) Pie diagram breaking down all network interactions into directed (from SignaLink, KEGG Pathway, and WikiPathways databases) and undirected interactions (STRING and HuRi). (b) Fraction of directed and undirected interactions in different network layers. (c) Fraction of directed and undirected interactions focusing on interactions between effectors and L1 proteins. Class 1, 4, and 12 effector interactions are dominated by information from SignaLink and the remaining classes are dominated by STRING.

## Slide 2
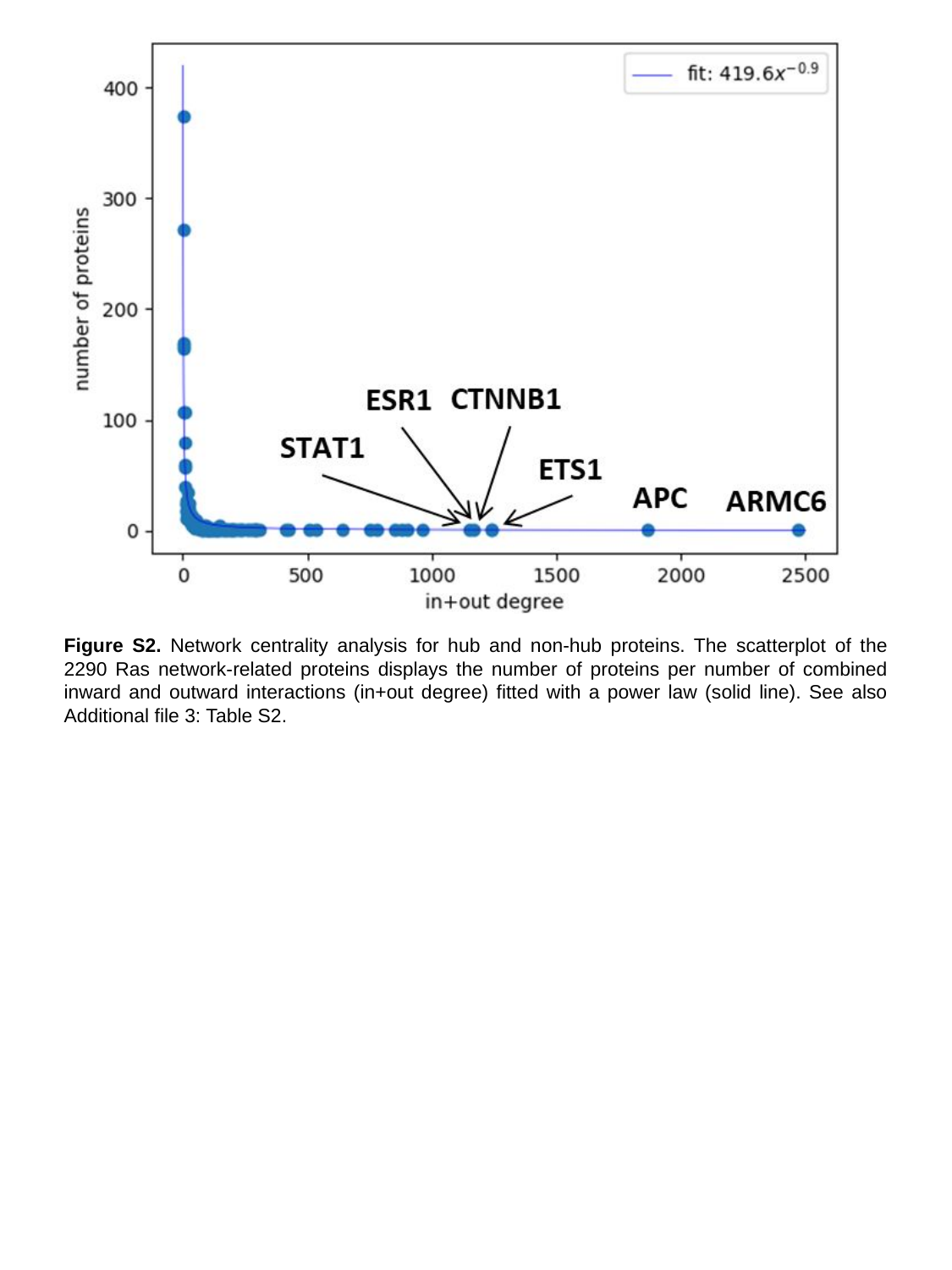

Figure S2. Network centrality analysis for hub and non-hub proteins. The scatterplot of the 2290 Ras network-related proteins displays the number of proteins per number of combined inward and outward interactions (in+out degree) fitted with a power law (solid line). See also Additional file 3: Table S2.

## Slide 3
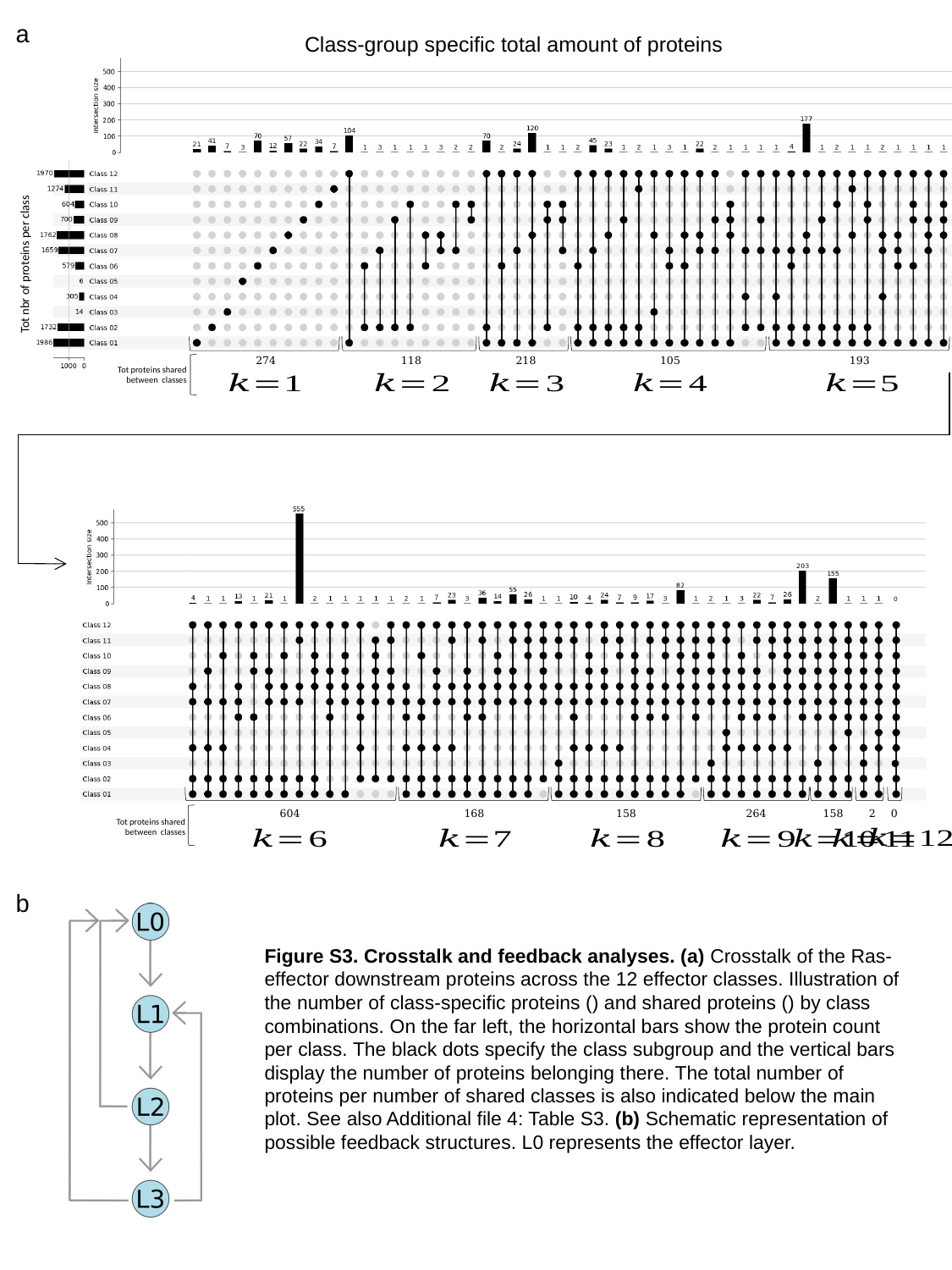

a
Class-group specific total amount of proteins
Tot nbr of proteins per class
274
118
218
105
193
0
158
2
0
604
168
158
264
b

## Slide 4
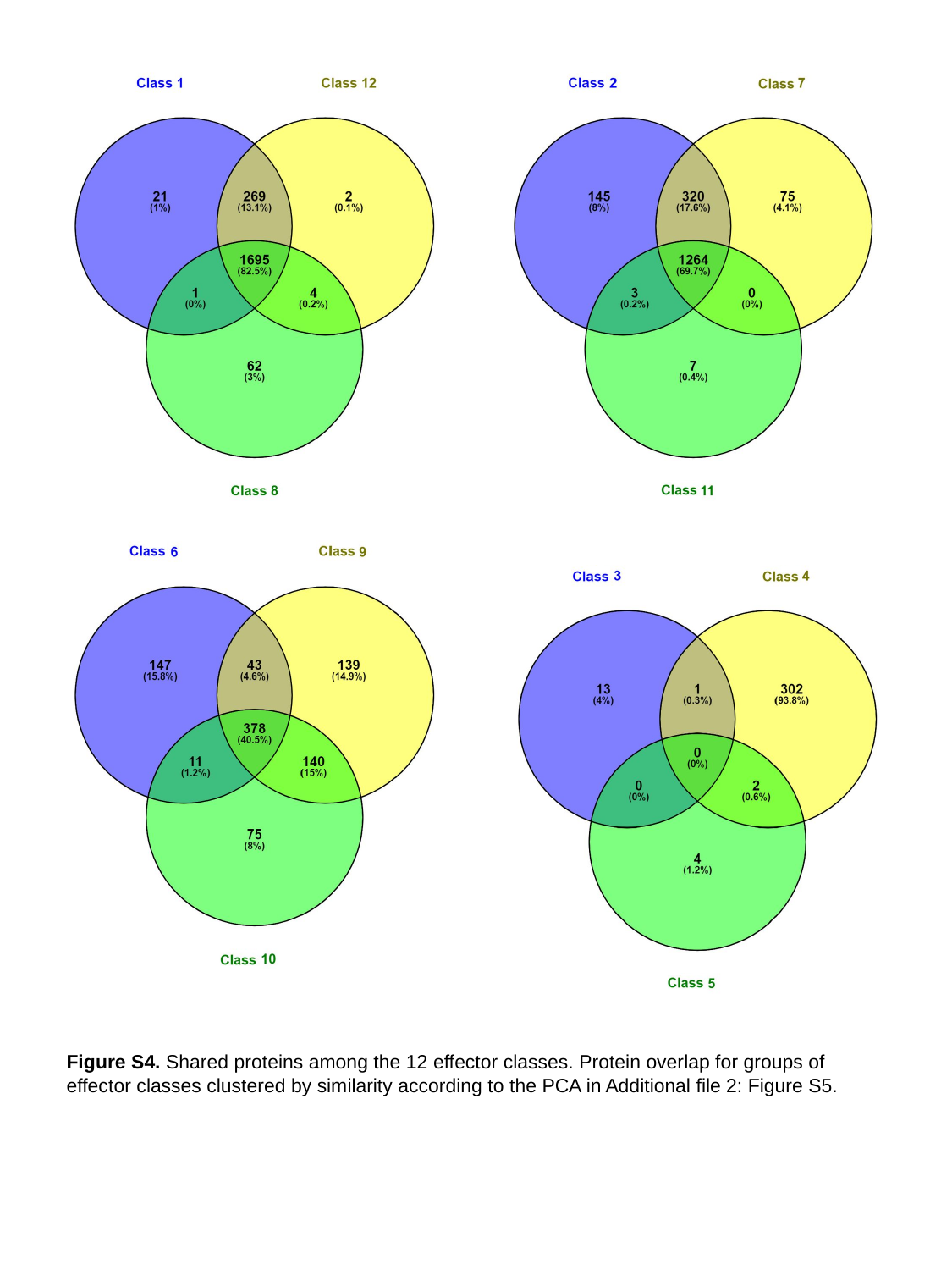

Figure S4. Shared proteins among the 12 effector classes. Protein overlap for groups of effector classes clustered by similarity according to the PCA in Additional file 2: Figure S5.

## Slide 5
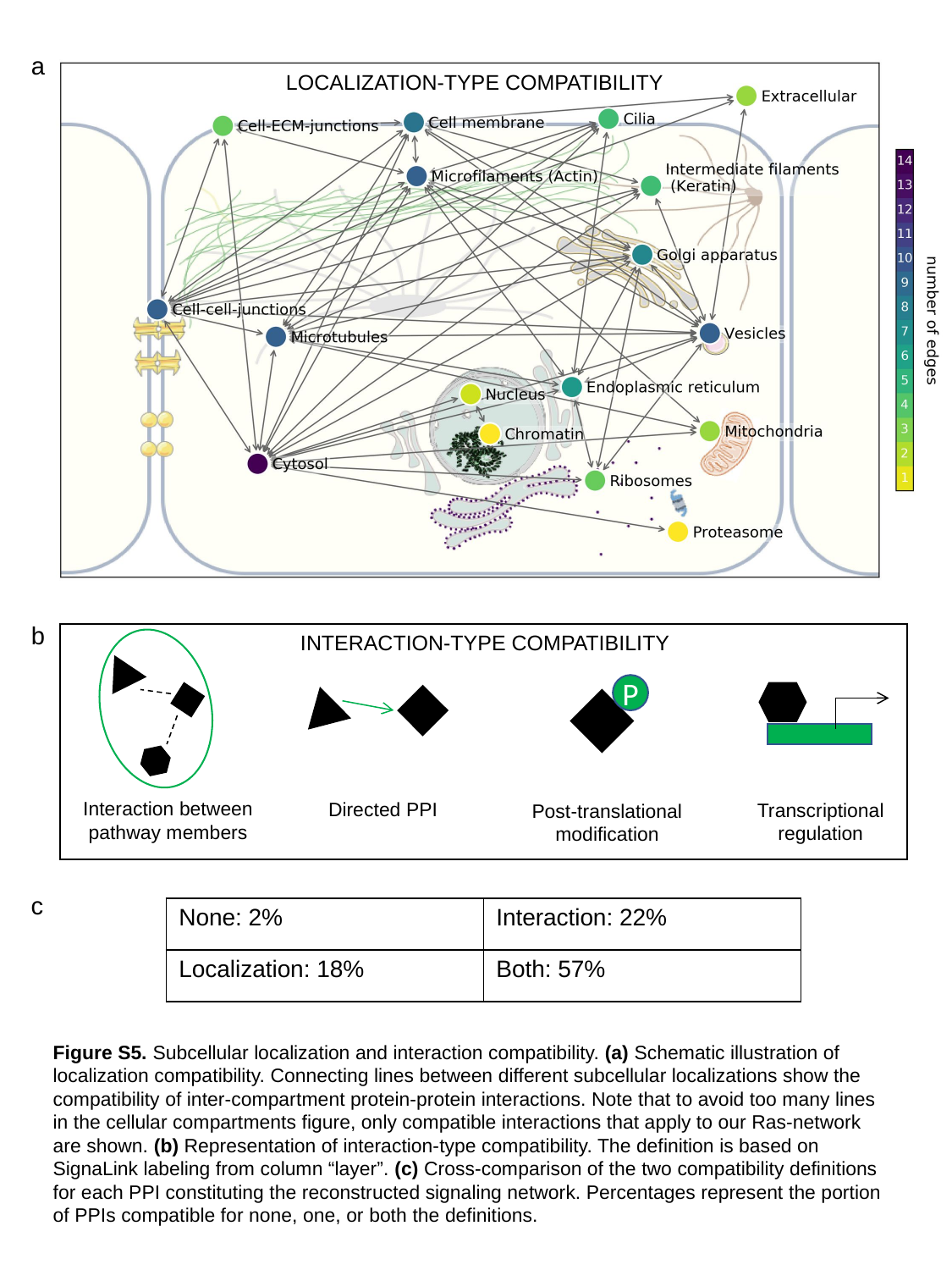

a
Localization-type compatibility
b
Interaction-type compatibility
P
Interaction between pathway members
Directed PPI
Transcriptional regulation
Post-translational modification
c
| None: 2% | Interaction: 22% |
| --- | --- |
| Localization: 18% | Both: 57% |
Figure S5. Subcellular localization and interaction compatibility. (a) Schematic illustration of localization compatibility. Connecting lines between different subcellular localizations show the compatibility of inter-compartment protein-protein interactions. Note that to avoid too many lines in the cellular compartments figure, only compatible interactions that apply to our Ras-network are shown. (b) Representation of interaction-type compatibility. The definition is based on SignaLink labeling from column “layer”. (c) Cross-comparison of the two compatibility definitions for each PPI constituting the reconstructed signaling network. Percentages represent the portion of PPIs compatible for none, one, or both the definitions.

## Slide 6
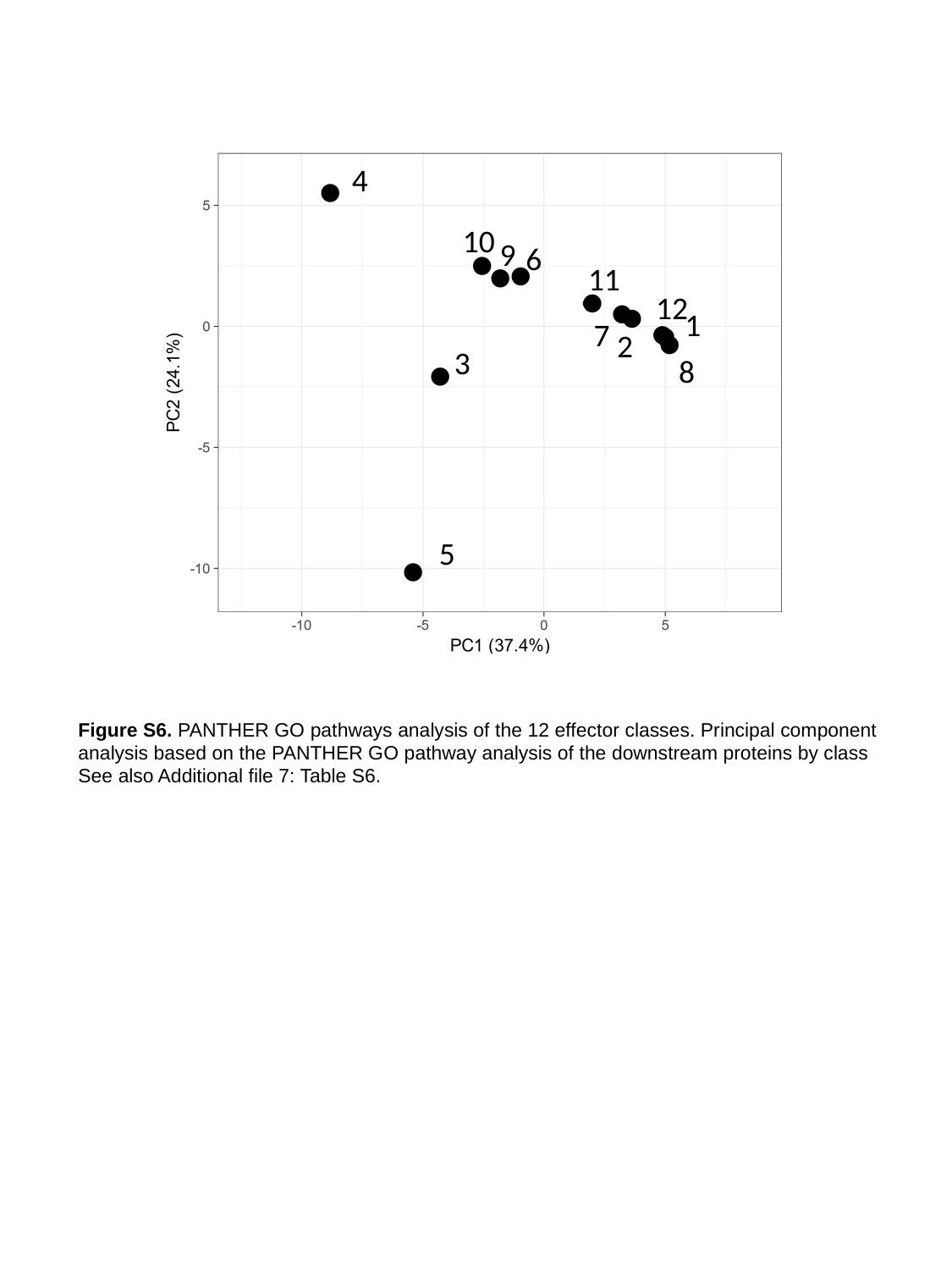

4
10
9
6
11
12
1
7
2
3
8
5
Figure S6. PANTHER GO pathways analysis of the 12 effector classes. Principal component analysis based on the PANTHER GO pathway analysis of the downstream proteins by class See also Additional file 7: Table S6.

## Slide 7
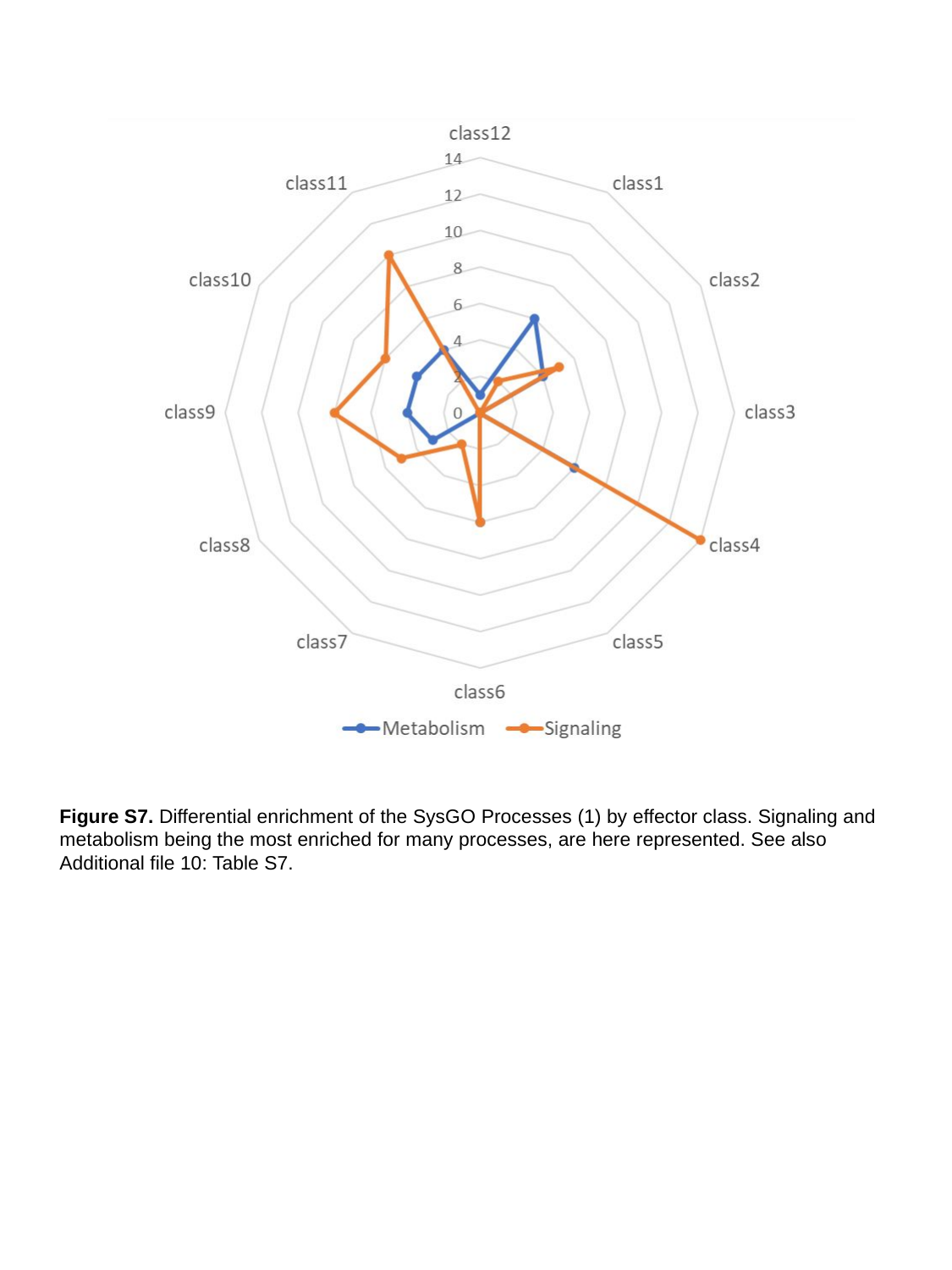

Figure S7. Differential enrichment of the SysGO Processes (1) by effector class. Signaling and metabolism being the most enriched for many processes, are here represented. See also Additional file 10: Table S7.

## Slide 8
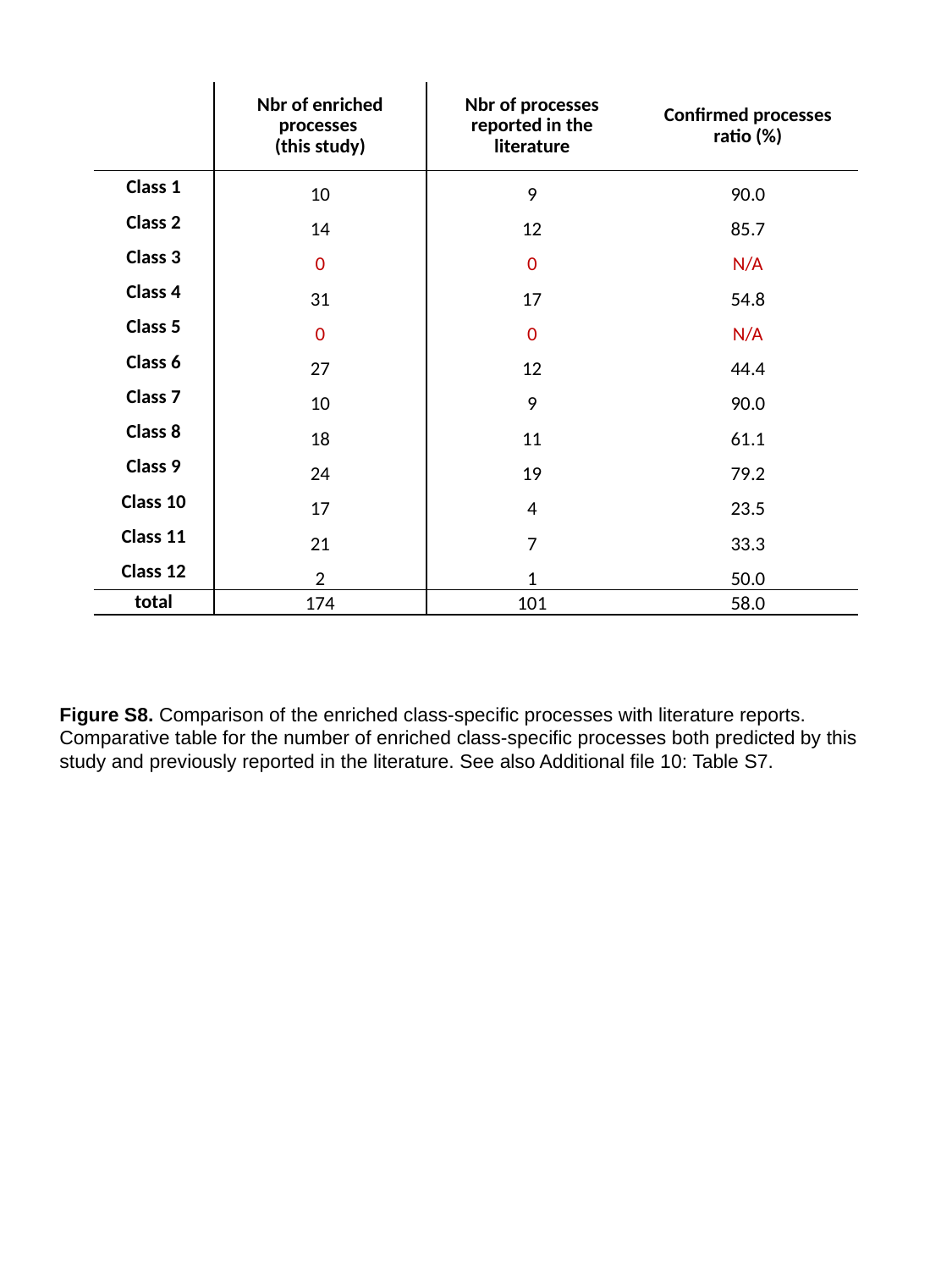

| | Nbr of enriched processes (this study) | Nbr of processes reported in the literature | Confirmed processes ratio (%) |
| --- | --- | --- | --- |
| Class 1 | 10 | 9 | 90.0 |
| Class 2 | 14 | 12 | 85.7 |
| Class 3 | 0 | 0 | N/A |
| Class 4 | 31 | 17 | 54.8 |
| Class 5 | 0 | 0 | N/A |
| Class 6 | 27 | 12 | 44.4 |
| Class 7 | 10 | 9 | 90.0 |
| Class 8 | 18 | 11 | 61.1 |
| Class 9 | 24 | 19 | 79.2 |
| Class 10 | 17 | 4 | 23.5 |
| Class 11 | 21 | 7 | 33.3 |
| Class 12 | 2 | 1 | 50.0 |
| total | 174 | 101 | 58.0 |
Figure S8. Comparison of the enriched class-specific processes with literature reports. Comparative table for the number of enriched class-specific processes both predicted by this study and previously reported in the literature. See also Additional file 10: Table S7.
