# Supplementary figures and images for: Reconstruction and analysis of a large-scale binary Ras-effector signaling network
Source: Cell Commun Signal. 2022 Mar 4;20:24. doi: 10.1186/s12964-022-00823-5 (PMC8896392; doi:10.1186/s12964-022-00823-5)

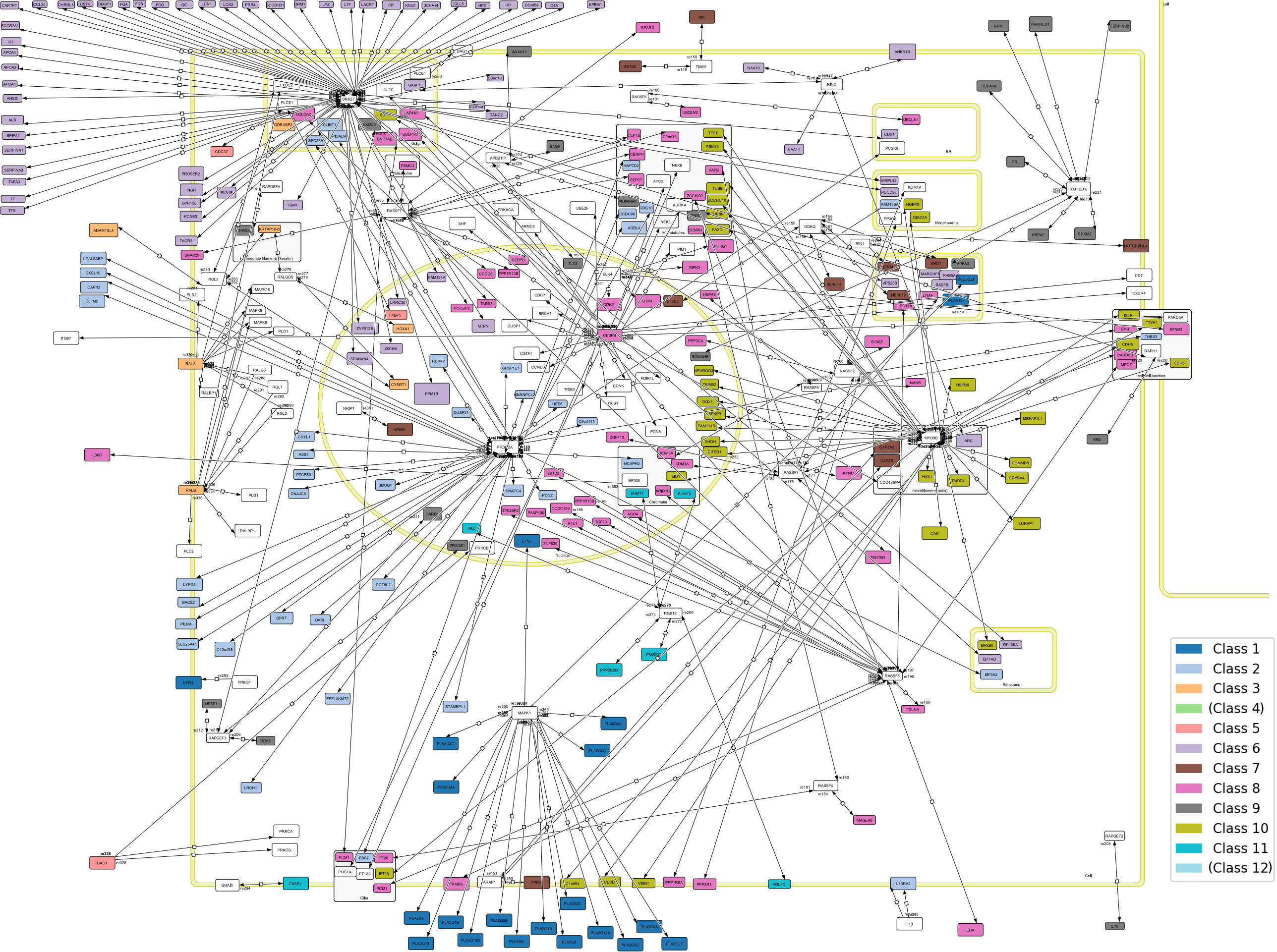

Supplement: Supplementary file 10 — Additional file 9: Network S2. CellDesigner diagram as pdf. [file 12964_2022_823_MOESM10_ESM.pdf]
